# Deep *R-gene* discovery in HLB resistant wild Australian limes uncovers evolutionary features and potentially important loci for hybrid breeding

**DOI:** 10.3389/fpls.2024.1503030

**Published:** 2025-01-29

**Authors:** Jianyang Liu, Khushwant Singh, Matthew Huff, Christopher Gottschalk, Michael Do, Margaret Staton, Manjunath L. Keremane, Robert Krueger, Chandrika Ramadugu, Chris Dardick

**Affiliations:** ^1^ Innovative Fruit Production, Improvement, and Protection, Appalachian Fruit Research Station, U.S. Department of Agriculture-Agricultural Research Service (USDA-ARS), Kearneysville, WV, United States; ^2^ Department of Botany and Plant Sciences, University of California, Riverside, Riverside, CA, United States; ^3^ Department of Entomology and Plant Pathology, University of Tennessee, Knoxville, TN, United States; ^4^ National Clonal Germplasm Repository for Citrus and Dates, U.S. Department of Agriculture-Agricultural Research Service (USDA-ARS), Riverside, CA, United States

**Keywords:** citrus, Honglongbing (HLB), R-genes, Australian limes, resistance

## Abstract

Huanglongbing (HLB) is a devastating citrus disease that threatens the citrus industry worldwide. HLB is associated with the bacteria *Candidatus Liberibacter asiaticus* (CLas) and as of today, there are no tools for economically viable disease management. Several wild Australian limes have been identified to be HLB resistant and their resistance is hypothesized to be conferred by resistance genes (R-genes), which mediate pathogen-specific defense responses. The aim of this study was to gain insight into the genomic features of R-genes in Australian limes, in comparison to susceptible citrus cultivars. In this study, we used five citrus genomes, including three Australian limes (*Citrus australasica*, *C. glauca* and *C. inodora*) and two cultivated citrus species (*C. clementina* and *C. sinensis*). Our results indicate up to 70% of the R-genes were identified in the unannotated regions in the original genome annotation of each species, owing to the use of a R-gene specific pipeline. Surprisingly, the two cultivated species harbored 15.8 to 104% more R-genes than the Australian limes. In all species, over 75% of the R-genes occurred in clusters and nearly 80% were concentrated in three chromosomes (Chr3, 5 and 7). The syntenic R-gene based phylogenic classification grouped the five species according to their HLB-resistance levels, reflecting the association between these R-genes and their distinct Australian origins. Domain structure analysis revealed substantial similarities in the R-genes between wild Australian limes and cultivated citrus. Investigation of chromosomal sites underlying Australian specific R genes revealed diversifying selection signatures on several chromosomal regions. The findings in this study will aid in the development of tools for genome-assisted breeding for HLB-resistant varieties.

## Introduction

Huanglongbing (HLB), also known as citrus greening, is a highly devastating disease that has affected many citrus-growing regions worldwide (Bové, 2006). HLB is associated with a phloem-limited bacterium, *Candidatus* Liberibacter asiaticus (*C*Las), which is transmitted through the Asian citrus psyllid (*Diaphorina citri*) or by grafting (Halbert and Manjunath, 2004). Infected citrus trees exhibit symptoms characterized by stunted and abnormal growth, reduced fruit quality and yield, tree decline, and death; there is no known cure for HLB (Anonymous, 2018), and no management strategies have proved to be truly effective in restoring infected trees (Li et al., 2021). Reduction in the rate of disease spread has been observed in regions such as California, which has implemented strict regulatory and operational guidelines to eradicate sources of inoculum (Garcia Figuera et al., 2022). However, such preventative strategies are insufficient to sustain the citrus industry in the long run (Bassanezi et al., 2020). The development of new citrus genotypes with desirable levels of tolerance/resistance to HLB would provide a long-term solution to the citrus industry.

Host resistance to HLB infection varies greatly across different citrus species and varieties. In examining the responses of 30 citrus genotypes to *C*Las inoculation, a wide range of resistance was observed across different genotypes (Folimonova et al., 2009). For instance, mandarin (*C. reticulata*) and sweet orange (*C. sinensis*) are more susceptible to HLB compared to Persian lime (*C. aurantiifolia*) or citrange (x *Citroncirus webberi*), in terms of both *C*Las titer and symptom severity (Folimonova et al., 2009). In evaluating HLB-resistance levels among 98 citrus accessions, Ramadugu et al. (2016b) developed eight HLB-resistance categories based on the qPCR results (i.e. cycle threshold values), intensity of HLB symptoms, and plant growth patterns. This system assigns a numeric code to each category of HLB-resistance, i.e. resistant plants (in category C1 and C2) support transient replication of *C*Las but lack pathogen establishment; tolerant plants (C3-C5) have delayed infection and can continue to produce fruits; susceptible plants (C6-C8) show typical disease symptoms including loss of foliage and die within four years. In the evaluated citrus accessions, some Australian limes were identified as resistant or tolerant, such as *C. glauca*, *C. australasica*, and *C. inodora*, whereas, many commercial cultivars were considered as susceptible, such as *C. sinensis* and *C. clementina*. These findings were confirmed in several greenhouse studies (Alquézar et al., 2021; Alves et al., 2021; Weber et al., 2022). A breeding program was initiated about ten years ago with the objective of introgressing HLB tolerance/resistance from wild Australian limes into commercial citrus (Ramadugu et al., 2016a, 2019; Ramadugu and Roose, 2024). Our understanding of the underlying mechanism of HLB pathogenesis would be greatly enhanced by identifying the target genes or quantitative trait loci (QTL) associated with HLB resistance.

Genotype-specific disease resistance in plants relies on the recognition of the pathogens by resistance genes (*R-genes*) that commonly encode a central nucleotide-binding site (NBS) domain and a C-terminal leucine-rich repeat (LRR) region that provides recognition specificity by interacting with the pathogen effectors (Moffett, 2009). The combination of NBS and LRR domains forms the NBS–LRR (NLR) structure, the core component in *R-genes* (McHale et al., 2006). NLR-containing genes can be further divided into two subfamilies, depending on the structure in the N-terminal domains, CC-NB-LRR (CNL) with a coiled-coil domain, and NIT-NB-LRR (TNL) with a Toll/Interleukin1 receptor domain (Elmore et al., 2011; Gururani et al., 2012). In addition to TNL and CNL, other major classes of *R-genes* include the RLKs (containing an extracellular LRR, a transmembrane domain and a cytoplasmic kinase domain), RLPs (which are similar to the RLKs but lack the kinase domain) and cytoplasmic enzymatic *R-genes* that contain neither LRR nor NBS groups (Gururani et al., 2012). Across plant species, *R-genes* are abundant and evolutionarily diverse. The abundance and diversity enable *R-genes* to detect a wide range of pathogens, followed by signaling cascades that lead to rapid defense responses, hypersensitive reactions, and programmed cell death (Dangl and Jones, 2001).

Given the essential role of *R-genes* in plant defense systems, exploration of their polymorphism across species can help gain insight into their resistance mechanisms. For example, by exploring *R-gene* regions in peanut, soybean, alfalfa, grape, and *Arabidopsis*, it was found that LRR domains underwent higher rates of duplication and DNA conversion compared to other regions, serving as the main contributor to evolution of resistance trait (Ratnaparkhe et al., 2011). In examining global expression patterns of *R-genes* in tomato and potato, some *R-genes*, such as NLR (required for cell death) were found to be expressed independent of infection status (von Dahlen et al., 2023). Comparison of genomic composition and structure of *R-genes* in cultivated and wild rice species unraveled the basis for the lack of resistance to bacterial blight in a rice cultivar ‘Kasalath’ (Mizuno et al., 2020). Similarly, a genome-wide comparative analysis of three citrus cultivars shed light on the structure, organization, and evolution of NBS genes in citrus genomes (Wang et al., 2015). However, no research has been conducted to systematically examine *R-genes* in multiple cultivated and wild citrus species, especially with regard to HLB tolerance/resistance.

Successful introduction of *R-genes* into plants usually relies on “NLR stacking”, the transferring of multiple *R-genes* on a single construct, to overcome the inability of a single locus to withstand high disease pressure (Zhang and Coaker, 2017). Resistance loci stacked with *R-genes* have been reported to be successful in several species. For example, two or three NLR loci were stacked in rice to provide resistance against rice blast (*Magnaporthe grisea*) (Fukuoka et al., 2015; Ellur et al., 2016). Three *Rpi* (resistance against *Phytophthora infestans*) genes have been stacked in potato simultaneously using a transgenic approach, resulting in robust resistance against late blight (Zhu et al., 2012). ATP binding cassette transporter and hexose transporter genes were introduced in wheat to prevent leaf rust (*Puccinia triticina*) and powdery mildew (*Blumeria graminis*), respectively (Krattinger et al., 2009; Moore et al., 2015). Accurate identification of resistance-providing *R-gene*(s) in citrus may be useful for molecular breeding and generating HLB-resistant transgenic plants.

Advancements in genetic and genomic technologies have enabled accurate identification of *R-gene* repertoires from diverse genotypes. *R-genes* are commonly found within arrays that are inherently repetitive or in regions with a high density of transposable elements (TEs) and are difficult to detect (Andolfo et al., 2022). Conventional pipelines usually annotate automatically predicted genes of the genome assemblies based on the search for *R-gene*-specific domains. Such approaches may be underperforming or imprecise, as repeat masking prior to automated genome annotation may preclude comprehensive *R-gene* detection (Andolfo et al., 2013; Jupe et al., 2013). Better accuracy and robustness in *R-gene* identification can be achieved using pipelines that can access sequences within and around highly repetitive regions (*e.g.* transposable elements and repeats) such as FindPlantNLR (Chen et al., 2023) which use genome as the starting point.

The genomes of wild Australian limes, with distinct evolutionary history and high levels of HLB-tolerance/resistance, are likely to harbor *R-genes* that are structurally and functionally different from those in the domesticated counterparts. Recent completion of the genome assembly and annotation of the three Australian limes (Singh et al., 2024) enables the identification and characterization of their *R-genes* as well as comparative analysis with commercial cultivars. Insights gained in such analyses would provide guidance to the breeding efforts in using Australian germplasm to introgress resistance-associated genes into cultivars for enhanced HLB tolerance/resistance.

## Material and methods

### Resources of genome sequences

In this study, we selected three Australian limes and two common citrus cultivars (**Table 1**). In the Australian limes, *C. glauca* was considered as HLB-resistant and in the HLB-resistance category C2, according to Ramadugu’s (Ramadugu et al., 2016b) evaluation system, whereas *C. australasica* and *C. inodora* were tolerant, in category C3. The two citrus cultivars *C*. *clementina* and *C*. *sinensis* were both susceptible and rated as C7. The chromosomal-scale genome assemblies of the three Australian limes are available at the NCBI Sequence Read Archive (SRA) (Singh et al., 2024), with their GenBank assembly numbers being GCA_029618585.1 (*C. australasica*), GCA_029633175.1 (*C. glauca*) and GCA_029721495.1 (*C. inodora*). The genome sequences of *C*. *clementina* and *C*. *sinensis* were downloaded from the Citrus Pan-genome to Breeding Database (http://citrus.hzau.edu.cn/orange/) (Wang et al., 2021; Wu et al., 2014), and their GenBank assembly numbers at NCBI are GCA_000493195.1 and GCA_019144185.1, respectively. These assemblies were of similar genomic sizes (298.9-376.5 Mb) and assembly quality with the scaffold N50 ranging from 28.9-37 Mb and scaffold L50 being 4 to 5. To ensure consistency of chromosome numbering between genomes, genomic alignment between *C. sinensis* and *C. clementina* was inspected using a web-based genome compare tool D-GENIES (Cabanettes and Klopp, 2018) (https://dgenies.toulouse.inra.fr). Chromosome numbers on *C. sinensis* were reassigned (for the present study) according to *C. clementina*, which was used as reference to order the scaffolds of the three Australian lime genome assemblies (Singh et al., 2024).

**Table 1:** Counts of annotated and classified *R-genes* in the genomes of three wild Australian limes (*C. australasica*, *C. inodora*, and *C. glauca*) and two cultivated c*itrus* species (*C. clementina* and *C. sinensis*).

Species	Resistance level	Genomic genes	NBARC genes	Masked R-genes	NLR			
					CNL	TNL	RNL	NL
***C. australasica***	C3	27348	616	479	174	110	3	213
***C. glauca***	C2	30067	564	402	114	105	4	226
***C. inodora***	C3	28173	404	154	109	69	3	138
***C. clementina***	C7	24534	689	257	205	104	3	267
***C. sinensis***	C7	29875	761	290	214	145	3	289

Total genes indicate the count of annotated genomic genes. The NBARC class contains genes with nucleotide-binding sites (NBS). The NLR class contains genes with NBS and Leucine-Rich Repeat (LRR) domains. The CNL, TNL and RNL classes all contain three domains, i.e., the two essential domains of NBS and LRR, and characteristic domains of Coiled Coil (CC), Toll-Interleukin receptor (TIR), and RPW8 (R), respectively. The NL in NLR class contain NBS, LRR and domains other than CC, TIR or RPW8. Masked *R-genes* are those that were newly identified in this study.

### Identification and classification of *R-genes* in wild Australian lime genomes

The primary haplotype assembly of the three Australian limes for each genome sequence was processed to remove any potential soft masking using in-house awk script (lowercase ACTGs that signify an annotated repeat or transposable elements). We downloaded the reference RefPlantNLR database (Kourelis et al., 2021) and the meme.xml file from the NLR-Annotator v.2 package (Bailey et al., 2015; Steuernagel et al., 2020). We executed the FindPlantNLR snakemake pipeline (Chen et al., 2023) to identify and annotate the NLR genes based on 13 Pfam accessions (**Supplementary Table S1**). The FindPlantNLR pipeline relies on tblastn, nhmmer, and NLR-Annotator to identify NLR loci (Camacho et al., 2009; Wheeler and Eddy, 2013; Steuernagel et al., 2020). The pipeline extended the potential region and 20 kb flanking sequence with bedtools (Quinlan and Hall, 2010). The extracted sequence was then input into the BRAKER annotation pipeline using the RefPlantNLR database as a model (Hoff et al., 2016). The resulting genes were scanned for motifs using Interproscan (Jones et al., 2014) followed by classification and script annotation within the FindPlantNLR package.

To determine which FindPlantNLR generated *R-genes* were retrieved from unannotated regions in the original genome annotation, we compared the NBARC annotation (.gff file) and the corresponding genome annotation using the gffcompare (v.0.9.12) software package within the GFF utilities (Pertea and Pertea, 2020). The results of *gffcompare* reported the matched and unmatched transcripts between the two annotation files. The *R-genes* that found no match in the genome annotation were considered as masked *R-genes*, i.e. they were not categorized as genes in the original genome annotation. The matched genes were categorized based on the types of matching relationship to reference transcripts and indexed by classification codes (detailed in **Supplementary Table S2**).

To compare the complement of *R-genes* identified in this study with previously reported *R-genes* in *C. sinensis* and *C. clementina*, we first made BLAST databases from the protein FASTA files of this study using *makeblastdb*. We compared the protein sequences of *R-genes* using *blastp* with HSP (high scoring pair) *e-value* set at 1e^-6^.

### Phylogenetic analysis, motif annotation, and chromosomal localization

Phylogenetic analysis of the five citrus species was performed on the protein sequences of the NBARC genes using the multiple sequence alignment program MAFFT (v7.526) (https://mafft.cbrc.jp/alignment) (Katoh and Standley, 2013) with the default setting. The phylogenetic relationship was visualized in a circular plot using the visualization program Chiplot (https://www.chiplot.online) (Xie et al., 2023).

### Species phylogeny based on BUSCO genes

For each citrus species genome, the BUSCO (v5.8.1) (Benchmarking Universal Single-Copy Orthologs) genes were retrieved and concatenated to generate an alignment supermatrix using a Python pipeline (https://github.com/jamiemcg/BUSCO_phylogenomics), which was used to construct species phylogeny using IQ-TREE (v2.3.6) (Nguyen et al., 2015) with 1000 bootstrap replicates. The tree structure was visualized using MEGA11 (Tamura et al., 2021).

### Synteny analysis of *R-genes*

Syntenic genes between each pair of citrus species were identified using the *One StepMCScanX-SuperFast* module in TBtools (v2.086) (Chen et al., 2020). Among these syntenic genes, 39 were identified to be common across all species and were used to construct phylogenetic trees following alignment and concatenation. The distribution and organization of these consensus syntenic genes were visualized using *Multiple Synteny Plot* in TBtools (Chen et al., 2020). Phylogeny based on these consensus syntenic genes was constructed using IQ-TREE (Nguyen et al., 2015) after alignment and concatenation. The syntenic genes within the genome of each species were analyzed in a similar way as described above and were visualized using the *Advanced Circos* module in TBtools (Chen et al., 2020). The clusters were defined by the presence of at least three genes that were located less than 200 kb apart (Holub, 2001).

### DNA sequence variation and Ka/Ks analysis

To estimate the selection pressure acting on *R-genes*, we calculated the rates of nonsynonymous substitution, synonymous substitution, and their rate ratio (Ka, Ks, and Ka/Ks) on each pair of syntenic *R-genes* between species using DnaSP6 (Rozas et al., 2017). Loci with calculated Ka/Ks values were plotted on chromosomes using the R package chromoMap (v0.4.1) (Anand and Rodriguez Lopez, 2022).

### Analysis of structural and functional differences between citrus genomes

The protein sequences were employed to infer structural and functional differences in *R-genes* between genomes using Orthovenn3 (Sun et al., 2023) with the OrthoMCL algorithm (E-value = 1e-2, inflation value = 1.5). Pairwise sequence similarities between species were calculated with a threshold of the e-value ≤ 1e^−5^, and the inflation value was set at 1.5 for orthologous cluster generation using the Markov clustering algorithm. The expansion or contraction in gene family sizes was analyzed using the CAFE5 (Mendes et al., 2021), which implements a birth-death model to infer phylogenetic history and evolutionary time. The biological processes and molecular functions associated with identified gene clusters were retrieved and identified with GO terms annotation. Based on the identified unique clusters, the unique genes were collected and used for GO enrichment analyses using the built-in feature in OrthoVenn3.

### Retrieval and analysis of *R-gene* domains

The sequences and coordinates of *R-gene* domains, including CC, TIR, and LRR, were retrieved using Hidden Markov model (HMM). Briefly, the HMM profiles using HMMER (v3.4) (http://hmmer.org/) were mapped on protein sequences (Singh et al., 2024) from *C. australasica*, *C. glauca*, *C. inodora*, *C. clementina* and *C. sinensis*. Accessions for CC, TIR, and LRR were retrieved from PFAM database version 36.0 (http://pfam.xfam.org/). Six LRR domains were successfully retrieved and designated as: LRR1 (PF00560.37), LRR3 (PF07725.16), LRR4 (PF12799.11), LRR5 (PF13306.10), LRR6 (PF13516.10), and LRR8 (PF13855.10). To ease the visualization of the domain organizations on *R-genes*, we curated 20 genes from each species that best represent the complete gene set of each species. In this process, we first retrieved and combined the sequences that contain all the *R-gene* domains using an in-house program (https://github.com/saikizu/DoBioPython), followed by alignment using Clustal Omega (v1.2.4) (Sievers et al., 2011). The alignments were used to construct phylogenetic trees using IQ-TREE (v2.3.4) (Minh et al., 2020) and the tree files (in Newick format) were then used in the python-based program Treemer (Menardo et al., 2018) to prune the leaf numbers down to 20 by eliminating those that contribute the least to the tree diversity. The domain composition and organization were visualized using *Simple Biosequence Viewer* in TBtools, v1.108 (Chen et al., 2020).

EMBOSS-CONS (v 6.6.0.0) (Madeira et al., 2024) was used to generate consensus sequences from the domain sequences in each species to compare the domains between species. In this process, a residue is considered to be consensus if the number of positive matches at the position is greater than half (≥3) of all the sequences in the alignment.

Motif detection was performed using the MEME SUITE (v5.5.7) (Bailey et al., 2015) using protein sequences of the NLR genes. Each sequence identified the top five motifs with a motif width between 6 and 50 amino acids. Identified motifs were concatenated, aligned, and subjected to phylogeny construction for comparison.

## Results

### R-gene classification

The D-GENIES (Cabanettes and Klopp, 2018) alignment was used to reassign chromosome numbers to genomes that were labelled differently to facilitate comparison between species. In the *de novo* assembled genomes of the three Australian limes, *C. clementina* was used as the reference for numbering the chromosomes. Mapping between *C. sinensis* and *C. clementina* identified 69.61% of matches with greater than 50% similarity between the two genomes (**Supplementary Figure S1A**). The dot plots (**Supplementary Figures S1B, C**) indicated five chromosomes (Chr1, 3, 4, 5, and 7) in *C. sinensis* were inconsistent with *C. clementina* and were relabeled (for the present study) as follows: Chr1→Chr7, Chr3→Chr5, Chr4→Chr1, Chr5→Chr3, Chr7→Chr4.

Using the R-gene specific annotation pipeline FindPlantNLR (Chen et al., 2023), we identified a wide range of NBARC genes in the five citrus species (**Table 1**). These NBARC genes accounted for about 1.4-2.5% of the total genes in each genome. The two cultivars harbored 11.8-88.4% more NBARC genes than each of the Australian limes. The lowest number of NBARC genes was found in C. inodora. Comparing the R-gene annotation with the original genome annotation, we found that FindPlantNLR uncovered many NBARC genes that were not predicted in the original gene annotation of each genome. Over 70% NBARC genes were not previously annotated in *C. australasica* and *C. glauca* and 30-40% had not been annotated in the other three species. For the NBARC genes that showed overlaps in the genome annotation, the matching relationship were categorized into 11 classes, in which three (coded as j, k, and o) represented more than 50% of all the NBARC genes (**Supplementary Table S2**). To evaluate the completeness of R-genes predicted in *C. clementina* and *C. sinensis* in this study, we compared the protein sequences of these newly annotated R-genes with the R-genes annotated in previous studies (Wang et al., 2015; Yin et al., 2023). The BLAST results indicated that the R-genes identified in this study contained the complements of previously reported R-genes in both species (**Supplementary Tables S3**-**S5**), with the average identity more than 94.8%.

Among the NBARC genes, most (78.9-86.2%) were found to harbor an LRR as well, thereby classified as NLR genes. Similar to NBARC genes, the two cultivars had 15.8-104% more NLR genes than each of the three Australian species, and, *C. inodora* had the fewest. About 50% of NLR genes in each species fell into the CNL or TNL category, both of which varied highly between species. While the numbers were similar in *C. glauca*, the CNL genes were nearly two times higher than that of TNL genes in *C. clementina* and 47.5-63.8% higher in other species. With a few NLR genes identified as RNL, the rest of the NLR genes were classified as NL, which accounted for about 50% of the total NLR genes.

### Phylogenetic inferences

**Figure 1:**
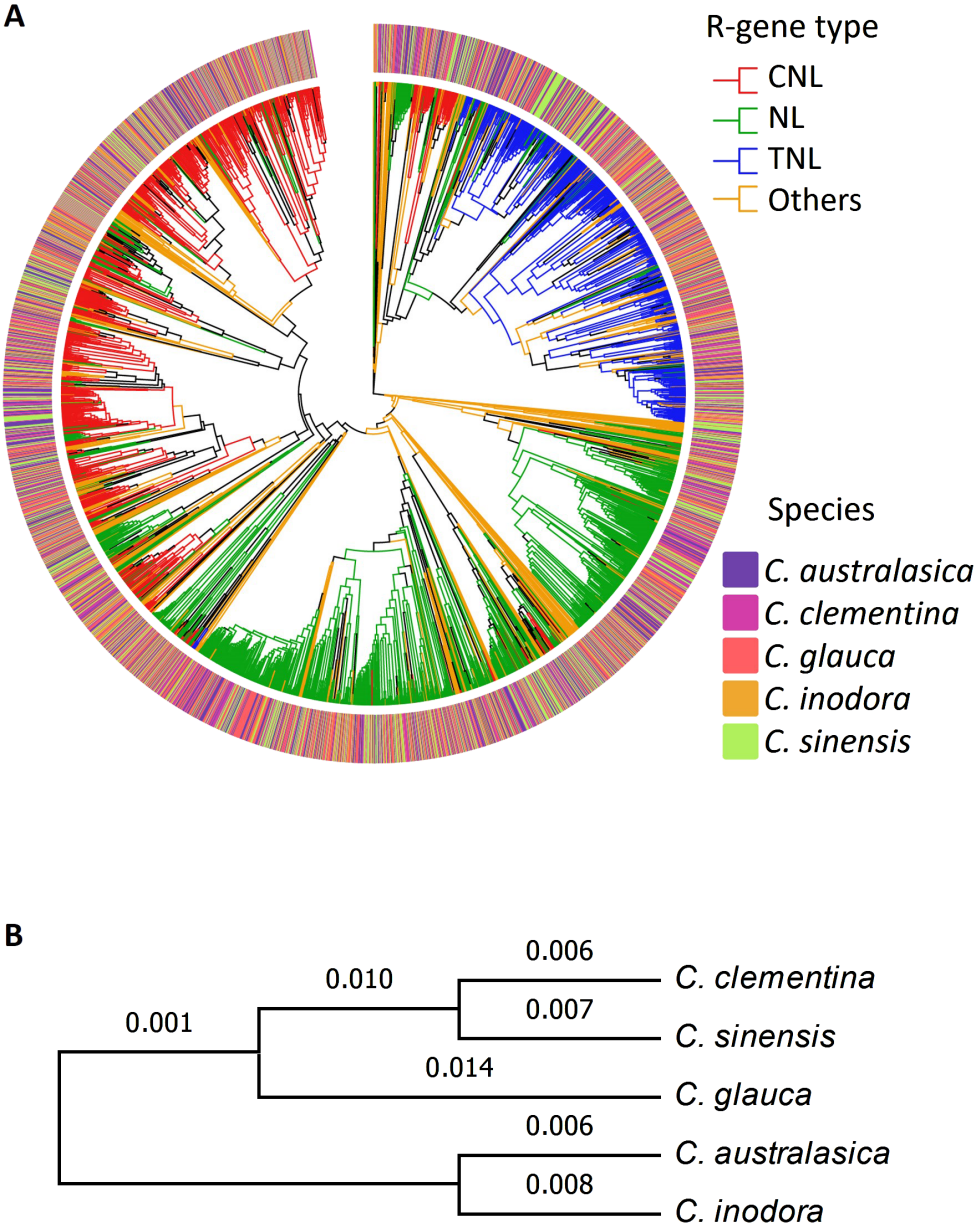
**(A)** Phylogenetic tree of the five citrus species based on NLR genes. **(B)** Phylogenetic tree of five citrus based on BUSCO genes (numbers on each branch are support values).

Phylogenetic analysis using protein sequences of the NBARC genes of the five citrus species generated three major clades (**Figure 1A**), which corresponded to three major *R-gene* families, namely CNL (red), TNL (blue) and NL (green). Other NBARC type of genes (orange) formed small clusters and dispersed among the phylogenic branches. In contrast to the distinct clades between *R-gene* classes, the relatedness of the species were not adequately depicted, and there was no distinction between the three Australian species, *C. australasica* (purple), *C. inodora* (orange) and *C. glauca* (red), and the two cultivated species of *C. clementina* (pink) and *C. sinensis* (green).

Using the protein sequences, we first explored the relationship between the five citrus species on a whole genome scale. We identified and aligned all single-copy BUSCO genes in all the genomes and constructed a robust maximum likelihood phylogenetic tree (**Figure 1B**). The phylogeny grouped the five species into two major clades, with *C. australasica* and *C. inodora* forming one clade and the other three species forming the other clade, in which the two cultivars form a sub-clade. It’s noticeable the support values on most branches were low, signifying a lack of strong distinction between the clades represented by the BUSCO genes.

### *R-genes* syntenic between and within genomes

To identify the conservation of homologous genes and R gene organization between the genomes of these species, a comprehensive pairwise synteny analysis was conducted using MCScan (**Supplementary Figure S2**). Our analysis revealed extensive synteny as *R-genes* were largely conserved between species. For example, 88.1% of all the NLR genes in *C. inodora* (*Cin*) were syntenic to *C. sinensis* (*Csi*), and on average, 53.2% of *R-genes* in each genome are syntenic with those in another species. On the other hand, the synteny also reflected the accumulation of structural variations, such as rearrangement and translocation, as displayed in the pairs of *C. australasica - C. glauca* (Cas-Cgl), *C. australasica - C. inodora* (Cas-Cin) and *C. sinensis - C. glauca* (Csi-Cgl). It should be noted that no distinct structural divergence was identified between the *R-genes* in Australian limes and the two cultivated citrus species.

**Figure 2:**
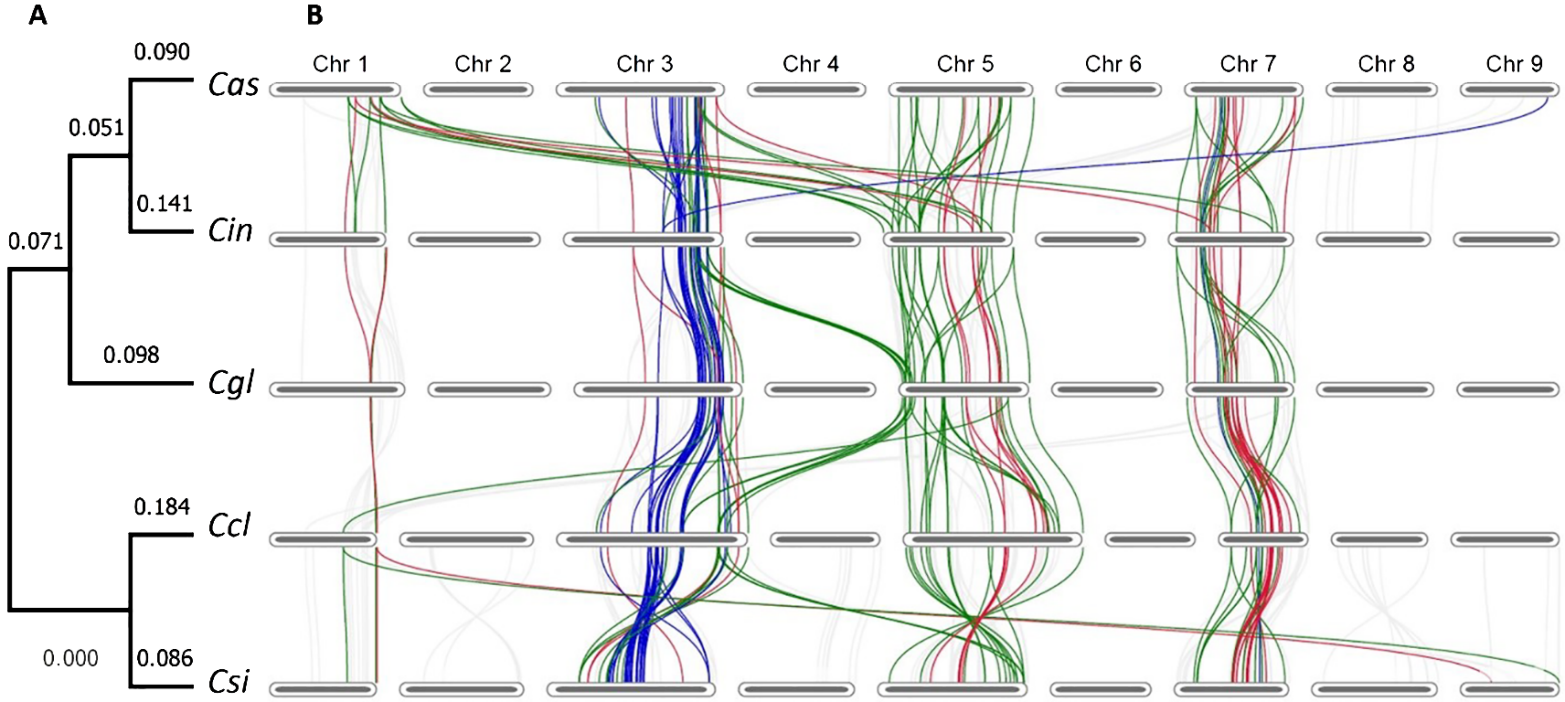
**(A)** Phylogenic tree of five citrus species based on 39 shared syntenic *R-genes*. Species names are abbreviated as follows *Cas*, *C. australasica*; *Cin*, *C. inodora*; *Cgl*, *C. glauca*; *Ccl*, *C. clementina*, and *Csi*, *C. sinensis*. **(B)** Chromosome-scale synteny of *R-genes*, with the 39 *R-genes* highlighted (chromosome sizes not depicted to scale) and *R-gene* classes color coded (NL = green, CNL = red, TNL = blue, and others = grey).

Among all the syntenic *R-genes* identified in pairwise comparisons, 39 were found to be common across all five species. With these common syntenic *R-genes*, we constructed a phylogenic tree, which properly grouped the five citrus species into clades according to their origins (**Figure 2A**). In this phylogeny, the two major clades represented the Australian limes and the cultivars. In the Australian lime clade, the two tolerant limes *C. australasica* and *C. indora* shared one clade and the resistant lime *C. glauca* was in a monotypic clade. It’s important to note that this phylogenic grouping was different from the phylogeny based on BUSCO genes, in which *C. glauca* was positioned in the cultivar clade. In these syntenic *R-genes*, the numbers of CNL (red), TNL (blue), and NL (green) genes were 10, 10, and 19, respectively (**Figure 2B**). Except for a large number of translocated genes positioned between Chr1 in *C. australasica* and Chr5 in *C. inodora*, most of these orthologs were located on the same chromosomes in each species, with nearly all TNL located on Chr3, most NL on Chr5, and most CNL on Chr7. Nearly all the TNL genes were found on Chr3, except one on Chr9 in *C. australasica*. In contrast, a few NL and CNL genes were located on different chromosomes.

Distinct patterns were found in chromosomal location of *R-genes* and the synteny of *R-genes* within each genome. The *R-genes* are distributed unevenly on the nine chromosomes in the five citrus species, with the majority of NLR genes residing on three chromosomes, Chr3, 5, and 7, in which Chr5 contained the most in each species (75.2-89.6%), followed by Chr3 and Chr7 (**Table 2**). Up to 75-89.4% of the NLR genes were found in clusters, i.e. at least three genes located within the range of 200 kb. Each of the two cultivars had more clustered NLR genes than the Australian limes. The average cluster size ranged from 7.7 to 10.8 genes, and the largest clusters varied greatly in size across species, ranging from 15 to 74 genes. For the NLR genes not in clusters, they occurred either in pairs or singles, and there were similar proportion of each (**Table 2**).

**Table 2:** Chromosomal distribution and clustering of NLR genes in five c*itrus* species.

Species	Chromosomal distribution (%)				Diversity in NLR genomic organization					
	Chr3	Chr5	Chr7	Others	Paired (%)	Singular (%)	Clustered (%)	No. of clusters	Size of largest cluster	Average cluster size
***C. australasica***	26.4	32.5	21.1	20.0	6.8	7.5	85.7	46	48	9.6
***C. glauca***	31.6	33.4	16.7	18.3	13.3	9.3	77.3	45	27	7.7
***C. inodora***	26.0	27.6	18.8	27.6	10.6	14.4	75.0	39	15	6.2
***C. clementina***	25.2	38.4	18.0	18.4	5.0	6.8	88.2	49	42	10.1
***C. sinensis***	28.1	31.6	22.3	18.0	4.6	6.0	89.4	54	74	10.8

Clusters were defined by the presence of at least three genes that were located in a 200 kb region.

**Figure 3:**
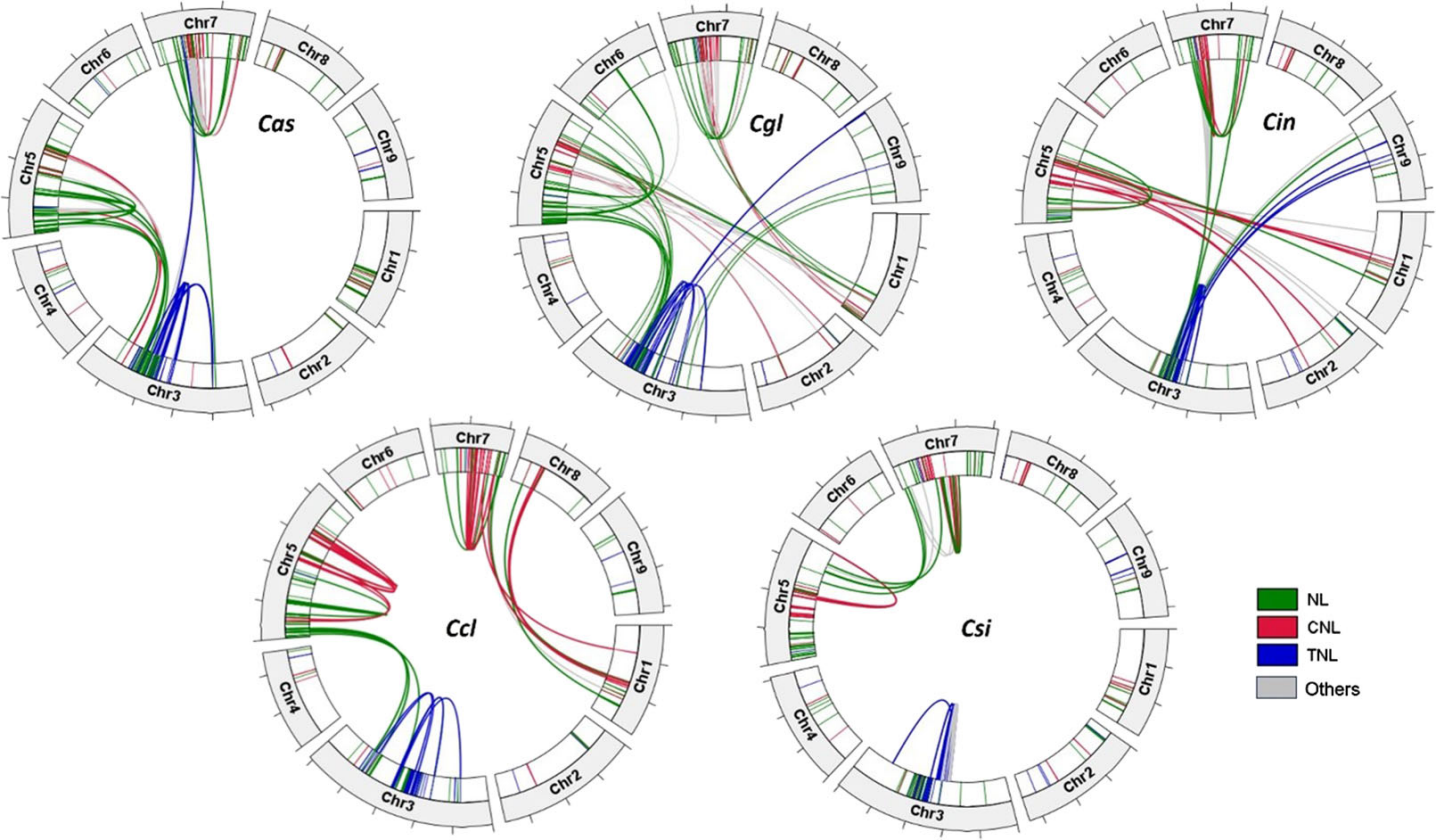
Circos plots of the *R-genes* in the genomes of five citrus species showing chromosomal locations, with link ribbons colored by *R-gene* classes (NL = green, CNL = red, TNL = blue, and others = grey). Each tick mark on the chromosome bars represents a 10-Mb interval. Species names are abbreviated as follows *Cas*, *C. australasica*; *Cin*, *C. inodora*; *Cgl*, *C. glauca*; *Ccl*, *C. clementina*, and *Csi*, *C. sinensis*.

On the three *R-gene* enriched chromosomes, *R-genes* of the same group tended to form clusters, residing in proximal regions on chromosomes in each species (**Figure 3**). In particular, nearly all TNL genes were located on Chr3 in tight clusters, whereas most CNL and NL*R-genes* resided either on Chr5 or 7. In *C. clementina* and *C. sinensis* genomes, TNL genes on Chr3 had syntenic genes exclusively on the same chromosome, while TNL genes on the Chr3 of the three Australian species all have syntenic genes on chromosomes other than Chr3. There are more inter-chromosomal syntenic pairs in Australian species compared to the two cultivars, especially *C. sinensis*, in which only three syntenic NL genes were found between Chr5 and 7. The tandem repeats of the NLR genes on the same chromosomes indicate tandem duplication, while the syntenies of genes from different chromosomes indicate duplication due to transposable elements or more complex rearrangements.

### Ka/Ks test on NLR orthologs

**Figure 4:**
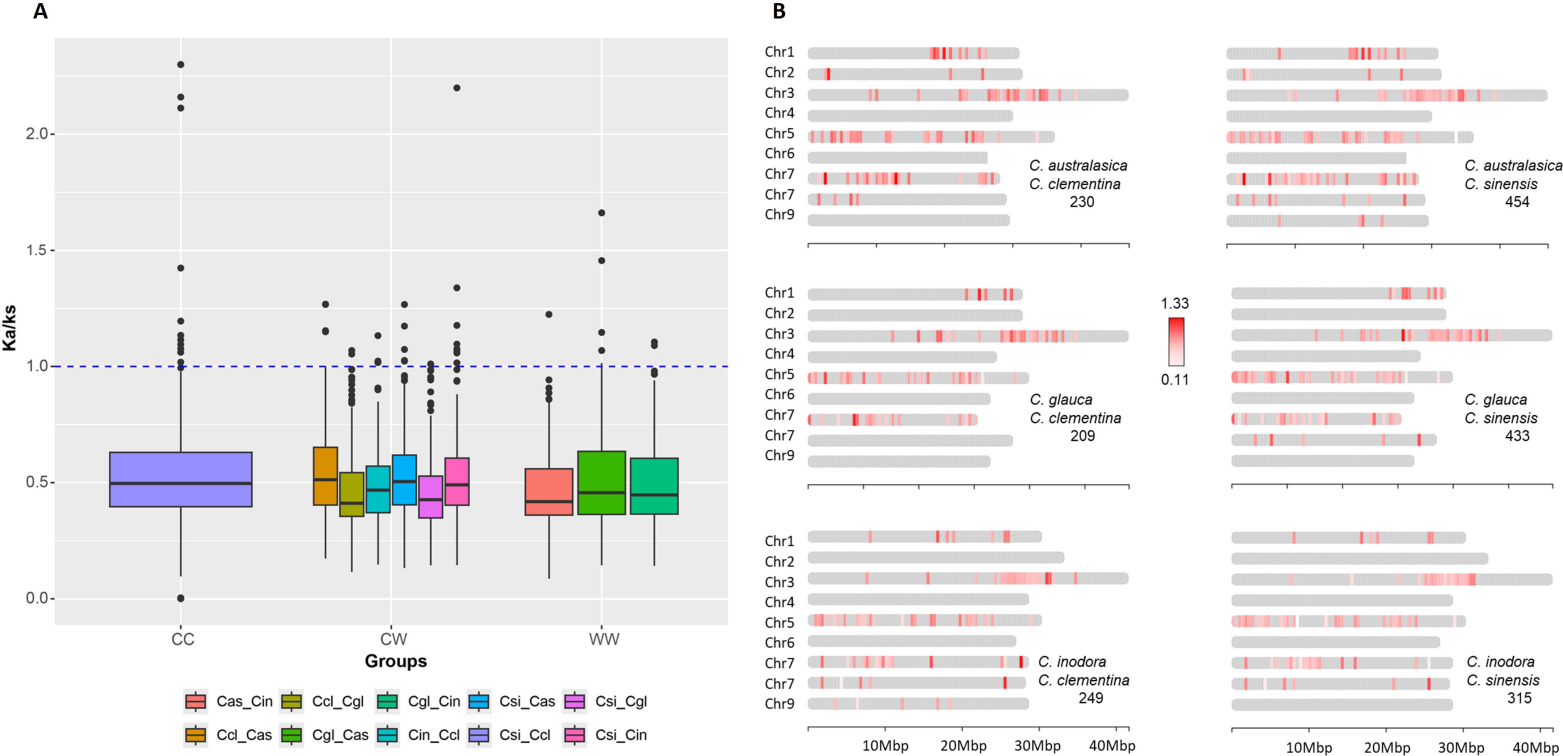
Ka/Ks analysis. **(A)** Box plot comparing the mean Ka/Ks ratios of orthologous gene pairs, within cultivars (CC), within Australian limes (WW) and between cultivars and Australian limes (CW). The horizontal bars inside boxes are median values. The box represents the interquartile range, between 25^th^ and 75^th^ percentiles. The whiskers represent smallest and largest values within 1.5 times interquartile range below 25th percentile and above 75th percentile, respectively. The circular dots represent outside values that are values beyond 1.5 times the interquartile range. Species names are abbreviated as follows Cas, *C. australasica*; Ccl, *C. clementina*; Cgl, *C. glauca*; Cin, *C. inodora*; Csi, *C. sinensis*. **(B)** Chromosomal distribution of orthologous *R-genes* with Ka/Ks values indicated by color intensity. Numbers of orthologous pairs are given under the pair of species.

To evaluate the effect of selective forces on the evolution of *R-genes* in these citrus species, we calculated the rates of nonsynonymous and synonymous substitution ratios (Ka/Ks) on each pair of syntenic *R-genes* between species using DnaSP6 (Rozas et al., 2017). The comparisons were made in three groups, with the first group (CC) including the two cultivars only, the second group including cultivars versus Australian species (CW), and the third group including the Australian species only (WW) (**Figure 4A**). In each group, the majority of the Ka/Ks ratios were less than 1, indicative of purifying selection. In the CC group, 12 genes have Ka/Ks ≥ 1, with the highest ratio reaching 2.26. In the CW group, there were 25 genes with Ka/Ks ≥ 1, with the comparison between having the most in *C. sinensis* vs *C. inodora*. There were seven genes with Ka/Ks ≥ 1 in the WW group.

The Ka/Ks ratios between the two cultivars ranged from 0 to 2.26, with the median being 0.495, and there are 12 genes that have Ka/Ks ≥ 1. In the comparison between cultivars and Australian limes, the median of the Ka/Ks ratios ranged from 0.41 to 0.51. There were 25 genes with Ka/Ks ≥ 1, and the highest ratio was 2.21 between *C. sinensis* and *C. inodora*. The comparison between Australian species showed seven genes with Ka/Ks ≥ 1, and the medians ranged from 0.41 to 0.45.

Mapping the Ka/Ks values in the CW group on the chromosomes depicted how selection acted differentially on the *R-gene* evolution between Australian species and cultivars (**Figure 4B**). The Ka/Ks values tended to be higher in the regions near the telomeres. Some regions on Chr5 of each comparison include low Ka/Ks values, suggesting these regions experienced strong purifying selection. Annotation of the 17 genes with Ka/Ks ≥ 1 between cultivars and Australian limes indicated that all the genes were related to ADP binding. Those from *C. clementina* were generally associated with coiled-coil domains, and the genes from the *C. sinensis* were all related to 10,13-epoxy-11-methyl-octadecadienoate biosynthesis (**Supplementary Table S6**).

### Orthologous cluster analysis and gene family evolution

**Figure 5:**
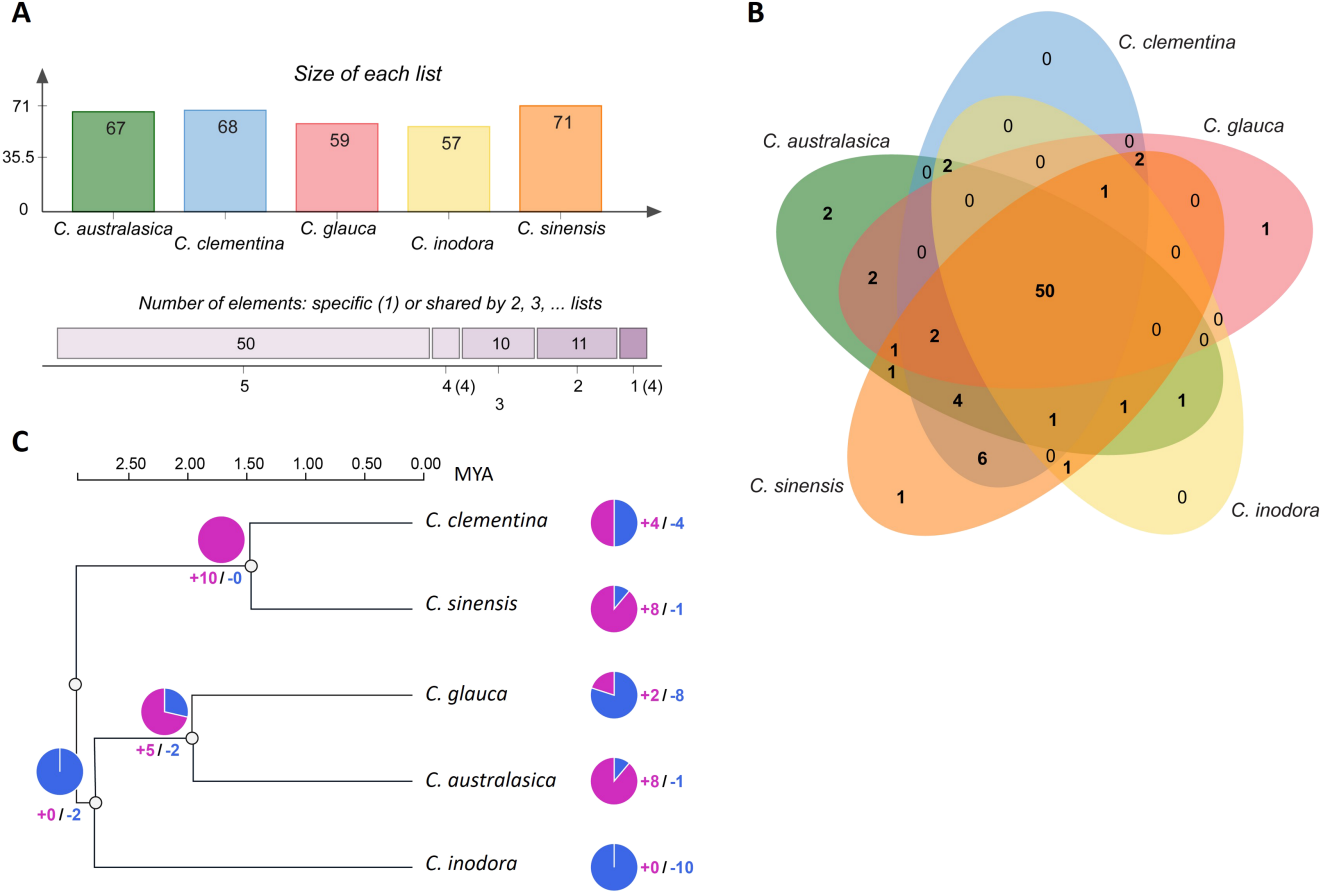
**(A)** Number of orthologous *R-gene* clusters from each species; **(B)** Venn diagram displaying orthologous *R-gene* clusters among five citrus species. **(C)** Phylogenetic relationship and divergence time based on protein sequences of orthologous *R-genes* from five citrus species, with dated nodes from Time-Tree. The divergence clock is indicated in million years ago (MYA) on the top. The numbers next to the pie charts represent the number of expanded (blue) or contracted (red) gene families.

Using Orthovenn3 (Sun et al., 2023), the collinearity analysis conducted for orthologous clusters on the *R-gene* protein sequences identified 57 to 71 orthologous clusters in the five citrus species (**Figure 5A**). About 50 orthologous clusters were shared by all five species, representing 92.8% of the total *R-genes* (**Figure 5B**). Two unique clusters were identified in *C*. *australasica*, comprising four genes; one unique cluster was found in *C*. *glauca* and *C. sinensis*, with two or three genes, respectively. No unique clusters were identified in *C. clementina* or *C. inodora*.

Gene family evolution analysis was employed to infer how *R-genes* diverged among these citrus species during the course of their evolution (**Figure 5C**). According to the dated phylogenetic tree on *R-genes*, the five species shared a common ancestry approximately 3 million years ago (MYA). The Australian limes underwent the first divergence around 2.75 MYA, in which *C*. *inodora* separated from *C. australasica* and *C. glauca*, which underwent another divergence of about 2.0 MYA. In contrast, the divergence between the progenitors of the two cultivars occurred 1.5 MYA.

All the *R-genes* in the three Australian limes belonged to one gene family and experienced gene family expansion approximately 3.0 MYA, followed by another gene family change of about 2.0 MYA, including contraction of 6 gene families and expansion of 2 gene families. In contrast, the first gene family change in the two citrus cultivars occurred around 1.5 MYA, in which all 11 gene families experienced contraction.

### Organization of *R-gene* domains in TNL genes

**Figure 6:**
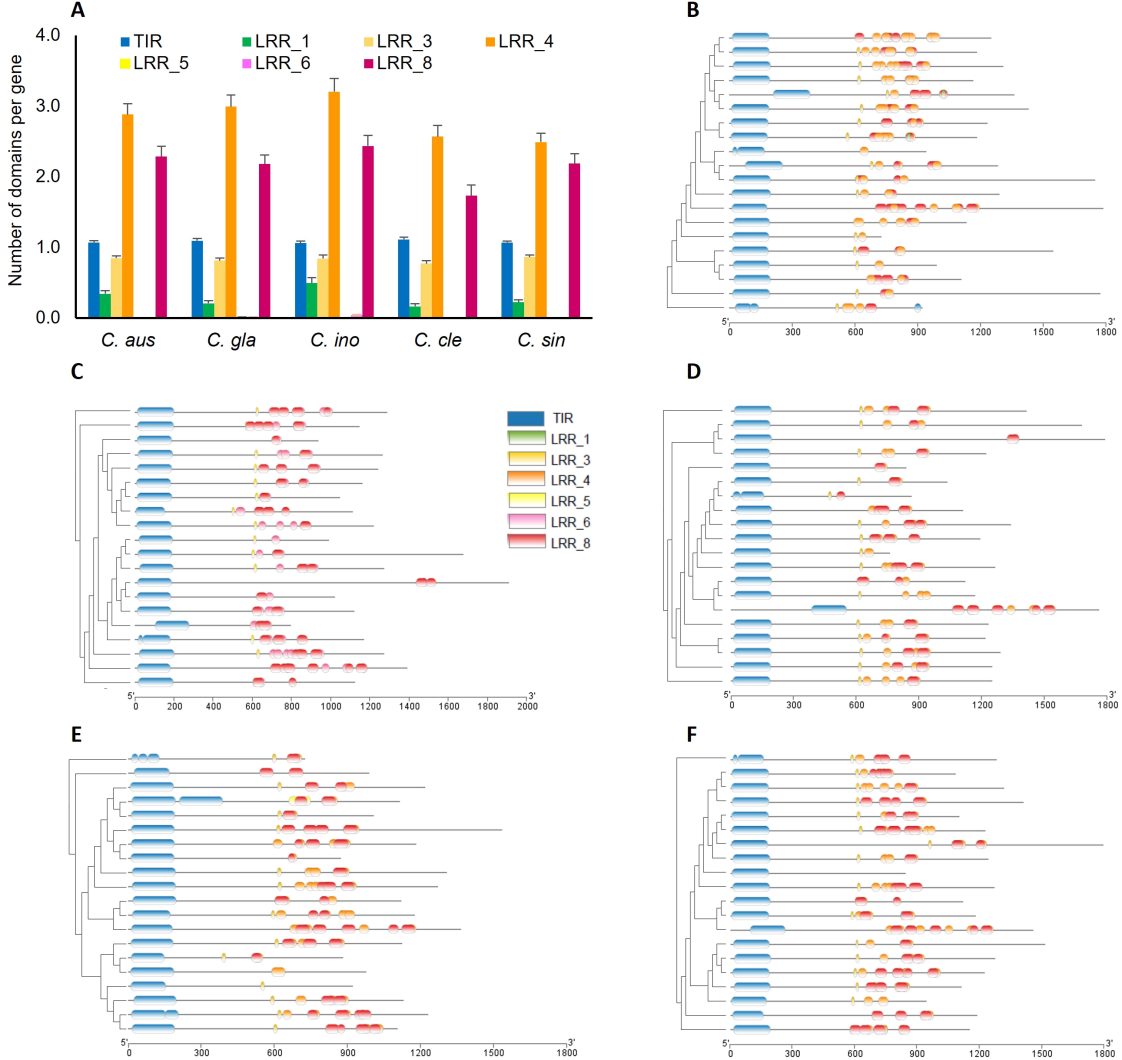
**(A)** Average number of domains per gene in each species (bars indicate *s.e*.). Species names are abbreviated as follows: *C. aus*, *C. australasica*; *C. ino*, *C. inodora*; *C. gla*, *C. glauca*; *C. cle*, *C. clementina*, and *C. sin*, *C. sinensis*. **(B–F)** Chromosomal distribution of TIR and LRR domains on 20 representative genes curated from each species.

To examine the composition and distribution of *R-gene* domains, we retrieved domain sequences and their chromosomal coordinates from TNL genes of each species. A similar composition of *R-gene* domains was found to reside on these genes across all the species (**Figure 6A**). In most genes, there was one single copy of full-length TIR and varying abundance of LRR domains. A total of six LRR domains were identified, with LRR1, 3, 4, and 8 common to all five species while LRR5, and LRR6 occurred rarely. The average numbers of the LRR4 domain (Pfam ID: PF12799) per gene were higher in the three Australian limes (2.9-3.2) compared to each of the two cultivars (2.5-2.6). The number of LRR8 (Pfam ID: PF13855) was lowest in *C. clementina*, and similar in the other four species. Domains of LRR1 and LRR3 only occurred in single copies on a gene, and there were, on average 0.29 and 0.83 per gene across the species.

To facilitate visualization and comparison of the chromosomal distribution of *R-gene* domains, we reduced the gene number in each species down to 20 using Treemer (Menardo et al., 2018), which evaluates the redundancy of phylogeny and only keeps the nodes that contribute the most to the phylogenetic diversity (**Figures 6B–F**). While TIR domains were generally located near the N-terminals in each gene, the LRR domains are 400-600 bp away, with most occurring in tandem arrangement. LRR4 and LRR8 are the dominant domains compared to the other two, and their copy numbers varied highly between genes, ranging from one to as many as 10. The distribution and organization of the LRR domains were highly variable in each species, and no clear patterns were demonstrated between resistant and susceptible types.

### Consensus sequences of LRR domains and motif analysis

**Figure 7:**
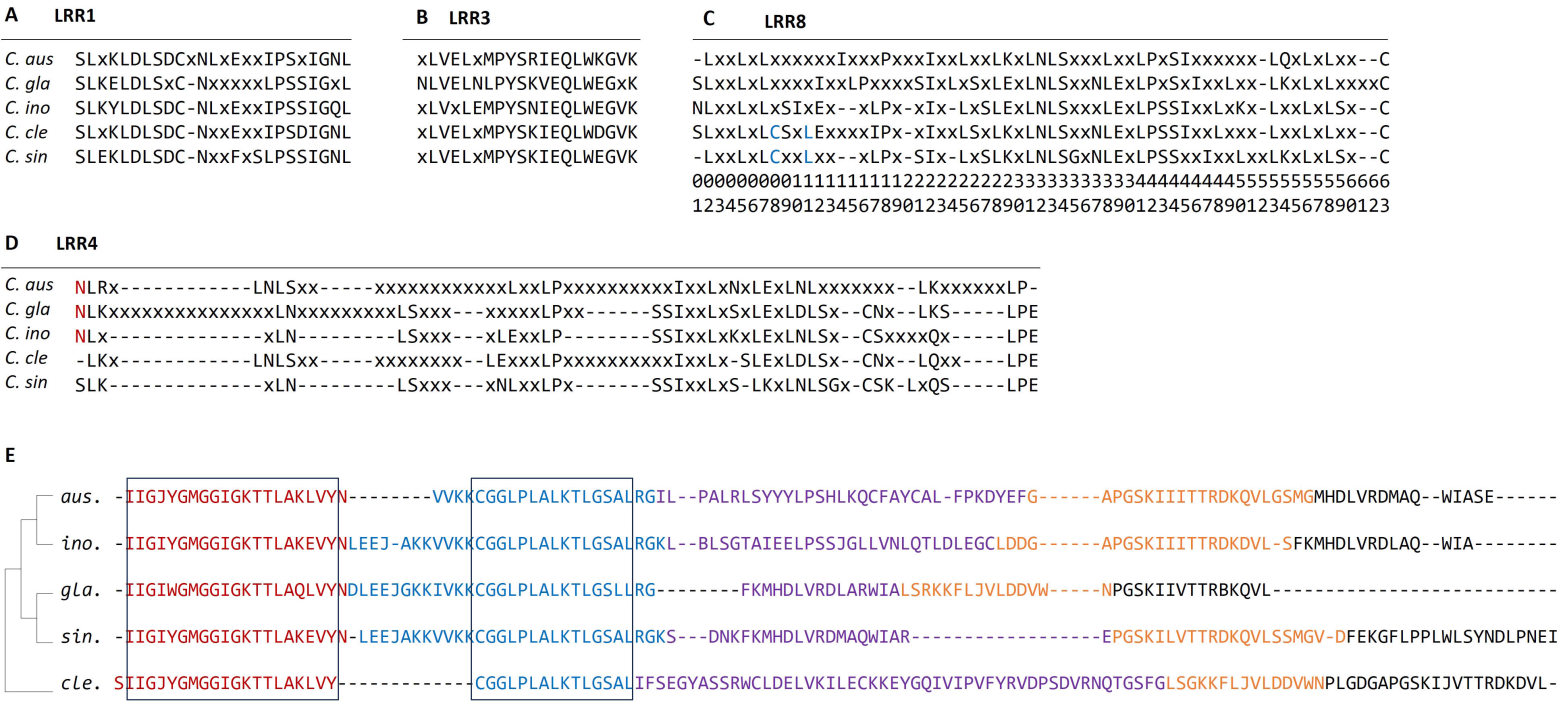
**(A**–**D)** Alignment of consensus sequences of four LRR domains from each species. Consensus residues are represented by 1-letter amino acid code; non-consensus residues are represented by “x” and gaps by “-”. The red letters indicate the common residue sequences that are specific to the Australian limes, and the blue letters indicate the common residue sequences specific to cultivated citrus species. **(E)** Alignment of top five conserved motifs from the five citrus species. The five domains are color coded by red (motif-1), blue (motif-2), purple (motif-3), orange (motif-4) and black (motif-5). The conserved regions are highlighted by black boxes. Species names are abbreviated as follows: *C*. *aus*, *C. australasica*; *C*. *ino*, *C. inodora*; *C*. *gla*, *C. glauca*; *C*. *cle*, *C. clementina*, and *C*. *sin*, *C. sinensis*.

The LRR domains in *R-genes* are often involved in specific recognition of pathogen effector molecules. Among the eight LRR domains identified in the five citrus species, four LRR domains (LRR1, 3, 4, and 8) were found to be common in each species. To facilitate comparison of the LRR domains between species, we employed EMBOSS-CONS (Madeira et al., 2024) to generate consensus protein sequences of each domain in each species (**Figure 7**). The alignment of the consensus sequences revealed that LRR1 and LRR3 are relatively short and highly conserved across the five species, with most positions residing with identical residues (**Figures 7A, B**). In contrast, the sequences in LRR4 are highly divergent, and there are many positions with non-consensus residues or gaps (**Figure 7D**). Except for *C. sinensis*, the other four species harbor several long stretches (≥5) of insertions composed of non-consensus residues missing in some species. In addition, the sequences of conserved positions are short and frequently interrupted by non-consensus residues. In the three Australian limes, the first positions are conserved, which all have an N-residue, but a gap and a serine residue in *C. clementina* and *C. sinensis*, respectively (**Figure 7D**, highlighted). The LRR8 domains are moderately conserved (**Figure 7C**). The two cultivars have two consensus residues at positions 8 and 11, which are non-consensus in the three Australian limes (**Figure 7C**, highlighted).

Motif analysis detects important molecular features, such as nucleotide binding sites, and predicts protein interaction domains on the target sequences. In this study, we identified the top five motifs from each *R-gene*, and most of these motifs are about 20 amino acids long (**Figure 7E**). The alignment of these motifs indicates the first domain was highly conserved among all the species, and the second motif is conserved in large regions of all five species. In contrast, no conserved segments were found in the other three motifs. The first two motifs are related to NBARC function, while the other three were not associated with any known function. According to concatenated motif sequences, phylogeny indicated that *C. sinensis* was closer to the Australian species than *C. clementina*.

## Discussion

In meeting the challenges of HLB devastation to citrus production, developing cultivars with sufficient tolerance/resistance to HLB has become imperative. Due to their natural tolerance/resistance traits, several researchers have used Australian limes toward this goal (Dutt et al., 2021; Weber et al., 2022). However, the underlying mechanism and genetic basis of the tolerance/resistance still largely remains unclear, thus hindering the breeding progress. Our aim was to investigate *R-genes* on the genomic level in the three Australian lime species in comparison with two major cultivated citrus varieties. Toward this end, we conducted multiple analyses on the five citrus species using a variety of analytical approaches to characterize their *R-gene* complements. Our results showed that the five taxa were phylogenetically distinct while sharing several highly conserved genomic regions.

### Total numbers of annotated and classified R-genes

In this study, we used a recently developed pipeline FindPlantNLR to comprehensively identify and annotate NBARC type *R-genes* from the five citrus species, including three wild Australian limes and two cultivated citrus species. This pipeline produces highly robust and reliable *R-gene* identification and annotation, enabling *R-gene* retrieval in unexplored genomic regions (Chen et al., 2023). As demonstrated in this study, FindPlantNLR uncovered many *R-genes* in each species that were unidentified in the original genome annotation. The proportion of the newly identified *R-genes* reached as high as over 70% in *C. australasica* and *C. glauca* and over 30% in other three species. In general, the NBARC loci cannot be accurately predicted by the automated gene annotation pipelines due to the repeat masking, a necessary step during genome assembly and gene annotation to avoid local genome assembly collapse and annotation errors (Tørresen et al., 2019). Since NBARC genes are typically organized in clusters of tandemly duplicated sequences, the *R-gene* loci are often obscured during genome annotation and inadvertently excluded from detection with the use of *R-gene* search pipelines that solely rely on predicted genes derived from genome annotation (Meyers et al., 2003; Andolfo et al., 2013). FindPlantNLRs overcomes this limitation by directly annotating the genome and may explain the discovery of high numbers of previously masked *R-genes* in this study. Our results show that this or similar pipelines that leverage direct annotation in unmasked genomes is essential for accurate and comprehensive prediction of the *R-genes* in plants.

In the two cultivars *C. sinensis* and *C. clementina*, we identified 689 and 761 NBS genes from the genomes, respectively, numbers higher than the previously reported *R-genes*. For example, only 111 NBARC domains were identified in protein sequences of *C. sinensis* (Yin et al., 2023). Combining open reading frames (ORFs) search and protein sequences selection resulted in prediction of higher numbers of NBARC genes in *C. sinensis* (Wang et al., 2016) than the earlier report (Yin et al., 2023), but still significantly lower than our prediction. Further, only about 50% of NBARC genes in the study by Wang et al. (Wang et al., 2015) could be classified as NLR genes, remarkably lower than our classification, ranging at 84-85%. Protein sequence comparison using PLASTP revealed that each NBARC gene identified in these studies was within the complement of *R-genes* predicted in our study in both species (**Supplementary Table S3**-**S5**). Considering the use of automatically annotated genomes in these studies, the above results reinforced the notion that approaches of direct genome detection are essential for more comprehensive and complete annotation of *R-genes*.

Comparing the overall R gene complements, the two cultivars harbored more NLR genes than the Australian limes, especially *C. inodora* and *C. glauca* (**Table 1**). It is not uncommon that the abundance of NBS genes in cultivated species is higher than in their wild relatives. For example, the numbers of NLR genes in the Asian cultivated rice (*Oryza sativa* L.) were found to be substantially higher than those in the wild ancestors (Mizuno et al., 2020), and cultivated soybean showed roughly 3-fold more TNL than its wild relative (*G. latifolia*) (Liu et al., 2018). Such *R-gene* expansion and duplication may have resulted from domestication and cultivation. Similar effects might also be at play in cultivated citrus, which are either natural hybrids or are bred mainly through interspecific hybrid and/or admixture (Wu et al., 2018). It has been suggested that retention of duplicated *R-genes* often incurs fitness costs, and as such natural selection tends to maintain a limited number of resistance genes in favor of more significant growth and reproduction. At the same time, domestication often enriches *R-genes* (Barabaschi et al., 2020).

Nevertheless, it should be noted that the abundance of *R-genes* in the genomes may not be directly related to resistance capability. As observed in this study, the Australian limes were found to have fewer NL*R-genes* than the cultivated species, though they have proved to be more tolerant/resistant to HLB (Ramadugu et al., 2016b). In the *R-gene* mediated defense system, the constitutive expression of a core set of *R-genes* is essential in implementing on-going defense status (von Dahlen et al., 2023), and different genotypes may vary extensively in the basal expression of most *R-genes*. For example, in a survey of 45 gene families studied in 19 accessions of *A. thaliana*, two *R-gene* subfamilies were found to be among the top three families of highly expressed genes, and the extent of differential expression for *R-genes* was surprisingly high, reaching up to 350-fold difference between accessions (Gan et al., 2011). Though *R-genes* are believed to function in a gene-for-gene manner, their expression patterns are also shaped by evolutionary paths and subjected to the influence of environmental factors (MacQueen and Bergelson, 2016), which may also explain the lack of association between *R-gene* abundance and resistance levels.

### *R-genes* are highly clustered

One striking feature of plant NBS-LRR genes are their frequent clustering arrangements. Our analysis showed NLR genes in each citrus species are preferentially mapped to three chromosomes (3, 5, and 7) and mainly occurred in clusters (**Table 2** and **Figure 3**). This result is consistent with previous findings in *C. sinensis* and *C. clementina*, in which 76.9 and 84.9% of the respective NBS genes were found in clusters (Wang et al., 2016). The high percentages of clustered NBS genes were also reported in other species such as rice (Yang et al., 2006), *Arabidopsis* (Guo et al., 2011), grapevine, and poplar (Yang et al., 2008). The synteny analysis indicated that the clustering of *R* genes may have arisen from both tandem duplication and translocation, which resulted in clustered sequences along or across chromosomes, respectively (**Figure 3**). The clustering arrangement is believed to be advantageous in providing coregulatory benefits and a broader detection spectrum against pathogens (van Wersch and Li, 2019). Accumulating evidence suggests the NLR genes continue to evolve in complexity and tend to function in higher-order configurations, such as NLR pairs or networks, formed by clustered NLRs, rather than dispersed genes or singletons (Contreras et al., 2023). For example, it has been found that coupled NLR genes are required to initiate resistance against a single pathogen in several species, such as genes associated with viral resistance in *Arabidopsis thaliana* and tobacco, leaf rust resistance in wheat, and blast resistance in rice (Zhai et al., 2014). The genetically clustered NLR genes in tight physical proximity supply raw genetic material for the acquisition of new resistance in the processes of sub-functionalization or neo-functionalization (Michelmore et al., 2013). The Australian wild limes included in the study originated from Australia where HLB disease or the associated pathogens have not been reported.

### HLB resistance levels reflected in phylogeny but not motifs

Phylogenetic relationships of *R-genes* depict the evolutionary connections of disease resistance between species. Using the 39 NLR genes that were common to each citrus species, we demonstrated that the phylogenetic inference precisely reflected their HLB-resistance levels, i.e. the three clades corresponded to the three resistance categories, i.e. resistant (C2) (containing *C. glauca*), tolerant (C3) (containing *C. australasica*, and *C. inodora*), and susceptible (C7) (containing *C. clementina*, and *C. sinensis*), based on Ramadugu’s (Ramadugu et al., 2016b) category system (**Figure 2**). This *R-gene* based phylogenetic inference was more accurate than the one using BUSCO genes (**Figure 1B**), which generated low support values on each branch and wrongly grouped an Australian lime (*C. glauca*) with the cultivated citrus species, suggesting *R-genes* may have evolved at a faster rate than the BUSCO genes. However, our domain structure and motif analyses (**Figures 6**, **7**) revealed no distinct patterns unique to either group, indicating resistance-related genomic characters may lie beyond the domains or motifs; the regulatory components may play an important role in determining resistance.

It has been demonstrated that *R-gene* expression is controlled by a complex yet tight regulatory system (Stokes et al., 2002; Holt et al., 2005; Li et al., 2007; Huot et al., 2014). The regulation of *R-gene* expression can operate on multiple levels, including transcriptional and epigenetic regulation, RNA interference (RNAi), splicing and translation, and post-translational controls (Kapos et al., 2019). In addition to the expression in the presence of pathogens, some *R-genes* were expressed variably across species irrespective of infection status (von Dahlen et al., 2023). Also, *R-gene* expression is highly tissue-specific, as evidenced in several transcriptomic studies (Chen et al., 2007; Zhai et al., 2014; Sharma et al., 2017). In addition, *R-genes* may be activated in response to environmental factors alone without biotic stimuli (MacQueen and Bergelson, 2016). Given the complexity of *R-gene* regulation, the HLB resistance observed in Australian limes may mainly be derived from regulatory mechanisms (in addition to the presence of essential *R-gene* domains).

### Loci with Ka/Ks values > 1

The Ka/Ks ratios, which measure the relative impact of diversifying and purifying selection on *R-gene* sites, were used to estimate which sites in the *R-genes* from the Australian limes were advantageous over cultivated citrus species. In the five Australian limes, we identified a total of 25 *R-genes* that had Ka/Ks ratios greater than one, indicative of positive selection in these sites. The low number of sites with high *Ka/Ks* ratios indicates limited evolutionary pressures in the Australian limes, suggesting the high conservation of the orthologous gene pairs between the two groups. Further, among these *R-genes*, 20 were located in clusters, and five existed as singletons. This finding is consistent with the proposal that *R-gene* singletons tend to maintain sequence stability and functional conservation under strong purifying selection, whereas *R-genes* in clusters undergo fast evolution to facilitate functional innovation in coping with changing pathogenic threats (Zhang et al., 2019). Similarly, in comparing cultivated citrus species, the median Ka/Ks rate ratio of singletons is less than those in clusters (Wang et al., 2015). Together, the *R-genes* identified in Australian limes may have accumulated beneficial mutations and may potentially serve as molecular markers to assist in breeding for pathogen-resistant cultivars.

### Evolution of R genes

The polymorphisms present within *R-genes* are subjected to the evolutionary forces. Fossil and molecular evidence has suggested that the genus citrus originated in southeast Asia, approximately 8 million years ago (Xie et al., 2023), and Australian citrus species arose during a major ancient species dispersal (Australian radiation) approximately 4 MYA (Schwartz et al., 2015; Wu et al., 2018). Chloroplast genome phylogeny (Wu et al., 2018) and pangenome analyses (Huang et al., 2023) indicated *C. glauca* diverged from *C. australasica* between 2-4 MYA. Our orthologous and phylogenetic analysis using NRL genes reflected the divergences between these species (**Figure 5C**), but on a relatively smaller time scale. This discrepancy may be due to the conservation in the *R-genes*, which are unlikely to show lineage sorting during rapid radiation and speciation events. The high overlapping of *R-gene* clusters also confirmed conservation among the *R-genes* in these species (**Figure 5A**). It is noticeable our result indicated the divergence between the progenitors of two cultivars were inferred to be 1.5 MYA, though selection of *C. clementina* was a recent event. This reflects some R-genes may have evolved at a faster rate, and thus inflated the estimation of evolutionary timeline between these two species.

### LRR structure may contribute HLB-resistance in Australian limes

The C-terminal LRR domains mediate pathogen recognition in NLR proteins through protein-protein interactions. As such, LRR domains contribute the most to *R-gene* polymorphism, as evidenced in the analysis of sequence mutations, protein secondary structure, and three-dimensional structures (Ratnaparkhe et al., 2011). In this study, we identified six LRR domains in the five citrus species, which exhibited high variation in abundance and genomic distribution (**Figure 6**). This result is consistent with the previous study in three citrus species, in which the LRR motifs showed high variation in sequences and repeat numbers (Wang et al., 2015). The sequence diversity in the LRR domains is consistent with the role of LRR domains in constituting a sensor domain that interacts with various molecular partners in detecting a variety of ever-evolving pathogens (Takkouche et al., 2023). Noticeably, LRR4 domain appeared to be more polymorphic between species compared to the other three LRR domains (**Figure 7**) and was more abundant in the three Australian limes than in each of the two cultivars (**Figure 6A**). Usually, one LRR domain detects a specific target effector, but plant LRR proteins must undergo conformational changes to induce the downstream defense responses (Liu et al., 2023). Therefore, the relative abundance of LRR4 in the Australian species may allow for expanded conformational complexity in mediating pathogen recognition, thus increasing regulatory capacity in coping with pathogenic attacks.

### Concluding remarks

In this study, we analyzed the genomic complement of *R-genes* in five citrus species to characterize the differences between Australian limes and cultivated species, thereby paving the way for the development of tools for genome-assisted breeding for HLB-resistant varieties. The syntenic analysis indicated the R-genes sequences contributed to the difference in HLB-resistance levels. However, substantial similarities in the genomic structure of *R-genes* were revealed in the five citrus species, and the identified polymorphisms were insufficient to distinguish between the two groups. These findings suggest that the HLB resistance in Australian limes may involve mechanisms other than R-genes. As suggested in a transcriptomic study (Weber et al., 2022), the resistance mechanisms in *C. australasica* may include phloem callose formation, redox control, phytohormone mediated signaling, secondary metabolites, secretion of pathogenesis-related (PR) proteins (i.e., Cys-rich secretory proteins and PR1-like proteins). Future investigations depicting association of HLB resistance and hybrids between resistant and susceptible citrus species in combination with RNA-seq data would yield more insights into *R-genes* along with other mechanisms that are responsible for HLB-resistance.

## Data Availability

The original contributions presented in the study are included in the article/**Supplementary Material**. Further inquiries can be directed to the corresponding author/s.

## References

[B1] Alquézar B. Carmona L. Bennici S. Peña L. (2021). Engineering of citrus to obtain huanglongbing resistance. Curr. Opin. Biotechnol. 70, 196–203. doi: 10.1016/j.copbio.2021.06.003 34198205

[B2] Alves M. N. Lopes S. A. Raiol-Junior L. L. Wulff N. A. Girardi E. A. Ollitrault P.. (2021). Resistance to ‘Candidatus Liberibacter asiaticus,’ the Huanglongbing Associated Bacterium, in sexually and/or Graft-Compatible Citrus Relatives. Front. Plant Sci. 11. doi: 10.3389/fpls.2020.617664 PMC7820388 33488659

[B3] Anand L. Rodriguez Lopez C. M. (2022). ChromoMap: an R package for interactive visualization of multi-omics data and annotation of chromosomes. BMC Bioinf. 23, 33. doi: 10.1186/s12859-021-04556-z PMC8753883 35016614

[B4] Andolfo G. Dohm J. C. Himmelbauer H. (2022). Prediction of NB-LRR resistance genes based on full-length sequence homology. Plant J. 110, 1592–1602. doi: 10.1111/tpj.15756 35365907 PMC9322396

[B5] Andolfo G. Sanseverino W. Rombauts S. Van de Peer Y. Bradeen J. M. Carputo D.. (2013). Overview of tomato (Solanum lycopersicum) candidate pathogen recognition genes reveals important Solanum R locus dynamics. New Phytol. 197, 223–237. doi: 10.1111/j.1469-8137.2012.04380.x 23163550

[B6] Anonymous (2018). The National Academies of Sciences: A Review of the Citrus Greening Research and Development Efforts Supported by the Citrus Research and Development Foundation: Fighting a Ravaging Disease (Washington, DC: The National Academies Press).

[B7] Bailey T. L. Johnson J. Grant C. E. Noble W. S. (2015). The MEME suite. Nucleic Acids Res. 43, W39–W49. doi: 10.1093/nar/gkv416 25953851 PMC4489269

[B8] Barabaschi D. Tondelli A. Valè G. Cattivelli L. (2020). Fitness cost shapes differential evolutionary dynamics of disease resistance genes in cultivated and wild plants. Mol. Plant 13, 1352–1354. doi: 10.1016/j.molp.2020.09.003 32916337

[B9] Bassanezi R. B. Lopes S. A. de Miranda M. P. Wulff N. A. Volpe H. X. L. Ayres A. J. (2020). Overview of citrus huanglongbing spread and management strategies in Brazil. Trop. Plant Pathol. 45, 251–264. doi: 10.1007/s40858-020-00343-y

[B10] Bové J. M. (2006). Huanglongbing: A destructive, newly-emerging, century-old disease of citrus. J. Plant Pathol. 88, 7–37. doi: 10.4454/jpp.v88i1.828

[B11] Cabanettes F. Klopp C. (2018). D-GENIES: dot plot large genomes in an interactive, efficient and simple way. PeerJ 6, e4958. doi: 10.7717/peerj.4958 29888139 PMC5991294

[B12] Camacho C. Coulouris G. Avagyan V. Ma N. Papadopoulos J. Bealer K.. (2009). BLAST+: architecture and applications. BMC Bioinf. 10, 421. doi: 10.1186/1471-2105-10-421 PMC2803857 20003500

[B13] Chen C. Chen H. Zhang Y. Thomas H. R. Frank M. H. He Y.. (2020). TBtools: an integrative toolkit developed for interactive analyses of big biological data. Mol. Plant 13, 1194–1202. doi: 10.1016/j.molp.2020.06.009 32585190

[B14] Chen R. Li H. Zhang L. Zhang J. Xiao J. Ye Z. (2007). CaMi, a root-knot nematode resistance gene from hot pepper (Capsium annuum L.) confers nematode resistance in tomato. Plant Cell Rep. 26, 895–905. doi: 10.1007/s00299-007-0304-0 17310335

[B15] Chen S. H. Martino A. M. Luo Z. Schwessinger B. Jones A. Tolessa T.. (2023). A high-quality pseudo-phased genome for Melaleuca quinquenervia shows allelic diversity of NLR-type resistance genes. GigaScience 12, giad102. doi: 10.1093/gigascience/giad102 PMC10720953 38096477

[B16] Contreras M. P. Lüdke D. Pai H. Toghani A. Kamoun S. (2023). NLR receptors in plant immunity: making sense of the alphabet soup. EMBO Rep. 24, e57495. doi: 10.15252/embr.202357495 37602936 PMC10561179

[B17] Dangl J. L. Jones J. D. G. (2001). Plant pathogens and integrated defence responses to infection. Nature 411, 826–833. doi: 10.1038/35081161 11459065

[B18] Dutt M. Mahmoud L. M. Chamusco K. Stanton D. Chase C. D. Nielsen E.. (2021). Utilization of somatic fusion techniques for the development of HLB tolerant breeding resources employing the Australian finger lime (Citrus australasica). PloS One 16, e0255842. doi: 10.1371/journal.pone.0255842 34375348 PMC8354479

[B19] Ellur R. K. Khanna A. Yadav A. Pathania S. Rajashekara H. Singh V. K.. (2016). Improvement of Basmati rice varieties for resistance to blast and bacterial blight diseases using marker assisted backcross breeding. Plant Sci. 242, 330–341. doi: 10.1016/j.plantsci.2015.08.020 26566849

[B20] Elmore J. M. Lin Z. J. Coaker G. (2011). Plant NB-LRR signaling: upstreams and downstreams. Curr. Opin. Plant Biol. 14, 365–371. doi: 10.1016/j.pbi.2011.03.011 21459033 PMC3155621

[B21] Folimonova S. Y. Robertson C. J. Garnsey S. M. Gowda S. Dawson W. O. (2009). Examination of the responses of different genotypes of citrus to huanglongbing (citrus greening) under different conditions. Phytopathology 99, 1346–1354. doi: 10.1094/PHYTO-99-12-1346 19900000

[B22] Fukuoka S. Saka N. Mizukami Y. Koga H. Yamanouchi U. Yoshioka Y.. (2015). Gene pyramiding enhances durable blast disease resistance in rice. Sci. Rep. 5, 7773. doi: 10.1038/srep07773 25586962 PMC5379001

[B23] Gan X. Stegle O. Behr J. Steffen J. G. Drewe P. Hildebrand K. L.. (2011). Multiple reference genomes and transcriptomes for Arabidopsis thaliana. Nature 477, 419–423. doi: 10.1038/nature10414 21874022 PMC4856438

[B24] Garcia Figuera S. Babcock B. Lubell M. McRoberts N. (2022). Collective action in the area-wide management of an invasive plant disease. Ecol. Soc. 27. doi: 10.5751/ES-13217-270212

[B25] Guo Y. L. Fitz J. Schneeberger K. Ossowski S. Cao J. Weigel D. (2011). Genome-wide comparison of nucleotide-binding site-leucine-rich repeat-encoding genes in Arabidopsis. Plant Physiol. 157, 757–769. doi: 10.1104/pp.111.181990 21810963 PMC3192553

[B26] Gururani M. A. Venkatesh J. Upadhyaya C. P. Nookaraju A. Pandey S. K. Park S. W. (2012). Plant disease resistance genes: Current status and future directions. Physiol. Mol. Plant Pathol. 78, 51–65. doi: 10.1016/j.pmpp.2012.01.002

[B27] Halbert S. E. Manjunath K. L. (2004). Asian citrus psyllids (Sternoarrhynca: Psyllidae) and greening disease of citrus: a literature review and assessment of risk in Florida. Florida Entomologist 87, 330–353. doi: 10.1653/0015-4040(2004)087[0330:ACPSPA]2.0.CO;2

[B28] Hoff K. J. Lange S. Lomsadze A. Borodovsky M. Stanke M. (2016). BRAKER1: unsupervised RNA-seq-based genome annotation with geneMark-ET and AUGUSTUS. Bioinformatics 32, 767–769. doi: 10.1093/bioinformatics/btv661 26559507 PMC6078167

[B29] Holt B. F. 3rd Belkhadir Y. Dangl J. L. (2005). Antagonistic control of disease resistance protein stability in the plant immune system. Science 309, 929–932. doi: 10.1126/science.1109977 15976272

[B30] Holub E. B. (2001). The arms race is ancient history in Arabidopsis, the wildflower. Nat. Rev. Genet. 2, 516–527. doi: 10.1038/35080508 11433358

[B31] Huang Y. He J. Xu Y. Zheng W. Wang S. Chen P.. (2023). Pangenome analysis provides insight into the evolution of the orange subfamily and a key gene for citric acid accumulation in citrus fruits. Nat. Genet. 55, 1964–1975. doi: 10.1038/s41588-023-01516-6 37783780

[B32] Huot B. Yao J. Montgomery B. L. He S. Y. (2014). Growth-defense tradeoffs in plants: a balancing act to optimize fitness. Mol. Plant 7, 1267–1287. doi: 10.1093/mp/ssu049 24777989 PMC4168297

[B33] Jones P. Binns D. Chang H. Y. Fraser M. Li W. McAnulla C.. (2014). InterProScan 5: genome-scale protein function classification. Bioinformatics 30, 1236–1240. doi: 10.1093/bioinformatics/btu031 24451626 PMC3998142

[B34] Jupe F. Witek K. Verweij W. Sliwka J. Pritchard L. Etherington G. J.. (2013). Resistance gene enrichment sequencing (RenSeq) enables reannotation of the NB-LRR gene family from sequenced plant genomes and rapid mapping of resistance loci in segregating populations. Plant J. 76, 530–544. doi: 10.1111/tpj.12307 23937694 PMC3935411

[B35] Kapos P. Devendrakumar K. T. Li X. (2019). Plant NLRs: From discovery to application. Plant Sci. 279, 3–18. doi: 10.1016/j.plantsci.2018.03.010 30709490

[B36] Katoh K. Standley D. M. (2013). MAFFT multiple sequence alignment software version 7: improvements in performance and usability. Mol. Biol. Evol. 30, 772–780. doi: 10.1093/molbev/mst010 23329690 PMC3603318

[B37] Kourelis J. Sakai T. Adachi H. Kamoun S. (2021). RefPlantNLR is a comprehensive collection of experimentally validated plant disease resistance proteins from the NLR family. PloS Biol. 19, e3001124. doi: 10.1371/journal.pbio.3001124 34669691 PMC8559963

[B38] Krattinger S. G. Lagudah E. S. Spielmeyer W. Singh R. P. Huerta-Espino J. McFadden H.. (2009). A putative ABC transporter confers durable resistance to multiple fungal pathogens in wheat. Science 323, 1360–1363. doi: 10.1126/science.1166453 19229000

[B39] Li X. Ruan H. Zhou C. Meng X. Chen W. (2021). Controlling citrus huanglongbing: green sustainable development route is the future. Front. Plant Sci. 12. doi: 10.3389/fpls.2021.760481 PMC8636133 34868155

[B40] Li Y. Yang S. Yang H. Hua J. (2007). The TIR-NB-LRR gene SNC1 is regulated at the transcript level by multiple factors. Mol. Plant Microbe Interact. 20, 1449–1456. doi: 10.1094/mpmi-20-11-1449 17977156

[B41] Liu Q. Chang S. Hartman G. L. Domier L. L. (2018). Assembly and annotation of a draft genome sequence for Glycine latifolia, a perennial wild relative of soybean. Plant J. 95, 71–85. doi: 10.1111/tpj.13931 29671916

[B42] Liu Q. Zhang C. Fang H. Yi L. Li M. (2023). Indispensable biomolecules for plant defense against pathogens: NBS-LRR and “nitrogen pool” alkaloids. Plant Sci. 334, 111752. doi: 10.1016/j.plantsci.2023.111752 37268110

[B43] MacQueen A. Bergelson J. (2016). Modulation of R-gene expression across environments. J. Exp. Bot. 67, 2093–2105. doi: 10.1093/jxb/erv530 26983577 PMC4793800

[B44] Madeira F. Madhusoodanan N. Lee J. Eusebi A. Niewielska A. Tivey A. R. N.. (2024). The EMBL-EBI Job Dispatcher sequence analysis tools framework in 2024. Nucleic Acids Res. 52, W521–w525. doi: 10.1093/nar/gkae241 38597606 PMC11223882

[B45] McHale L. Tan X. Koehl P. Michelmore R. W. (2006). Plant NBS-LRR proteins: adaptable guards. Genome Biol. 7, 212. doi: 10.1186/gb-2006-7-4-212 16677430 PMC1557992

[B46] Menardo F. Loiseau C. Brites D. Coscolla M. Gygli S. M. Rutaihwa L. K.. (2018). Treemmer: a tool to reduce large phylogenetic datasets with minimal loss of diversity. BMC Bioinf. 19, 164. doi: 10.1186/s12859-018-2164-8 PMC5930393 29716518

[B47] Mendes F. K. Vanderpool D. Fulton B. Hahn M. W. (2021). CAFE 5 models variation in evolutionary rates among gene families. Bioinformatics 36, 5516–5518. doi: 10.1093/bioinformatics/btaa1022 33325502

[B48] Meyers B. C. Kozik A. Griego A. Kuang H. Michelmore R. W. (2003). Genome-wide analysis of NBS-LRR-encoding genes in Arabidopsis. Plant Cell 15, 809–834. doi: 10.1105/tpc.009308 12671079 PMC152331

[B49] Michelmore R. W. Christopoulou M. Caldwell K. S. (2013). Impacts of resistance gene genetics, function, and evolution on a durable future. Annu. Rev. Phytopathol. 51, 291–319. doi: 10.1146/annurev-phyto-082712-102334 23682913

[B50] Minh B. Q. Schmidt H. A. Chernomor O. Schrempf D. Woodhams M. D. von Haeseler A.. (2020). IQ-TREE 2: new models and efficient methods for phylogenetic inference in the genomic era. Mol. Biol. Evol. 37, 1530–1534. doi: 10.1093/molbev/msaa015 32011700 PMC7182206

[B51] Mizuno H. Katagiri S. Kanamori H. Mukai Y. Sasaki T. Matsumoto T.. (2020). Evolutionary dynamics and impacts of chromosome regions carrying R-gene clusters in rice. Sci. Rep. 10, 872. doi: 10.1038/s41598-020-57729-w 31964985 PMC6972905

[B52] Moffett P. (2009). “Chapter 1 - mechanisms of recognition in dominant R gene mediated resistance,” in Advances in Virus Research. Eds. Loebenstein G. Carr J. P. (Amsterdam, Netherlands: Elsevier), 1–229. 10.1016/S0065-3527(09)07501-0 20109662

[B53] Moore J. W. Herrera-Foessel S. Lan C. Schnippenkoetter W. Ayliffe M. Huerta-Espino J.. (2015). A recently evolved hexose transporter variant confers resistance to multiple pathogens in wheat. Nat. Genet. 47, 1494–1498. doi: 10.1038/ng.3439 26551671

[B54] Nguyen L. T. Schmidt H. A. von Haeseler A. Minh B. Q. (2015). IQ-TREE: a fast and effective stochastic algorithm for estimating maximum-likelihood phylogenies. Mol. Biol. Evol. 32, 268–274. doi: 10.1093/molbev/msu300 25371430 PMC4271533

[B55] Pertea G. Pertea M. (2020). GFF utilities: GffRead and GffCompare. F1000Res 9, 304. doi: 10.12688/f1000research.23297.2 PMC7222033 32489650

[B56] Quinlan A. R. Hall I. M. (2010). BEDTools: a flexible suite of utilities for comparing genomic features. Bioinformatics 26, 841–842. doi: 10.1093/bioinformatics/btq033 20110278 PMC2832824

[B57] Ramadugu C. Keremane M. Lee R. F. Hall D. G. McCollum T. G. Roose M. L. (2019). Novel citrus hybrids with HLB resistance. Citrograph 10, 60–64. Available at: https://citrus-research-board-static.sfo2.digitaloceanspaces.com/citrograph/pdf/CRB-Citrograph-Mag-Q2-Spring-2019-Web.pdf (Accessed July 25, 2024).

[B58] Ramadugu C. Keremane M. L. Halbert S. E. Duan Y. P. Roose M. L. Stover E.. (2016b). Long-term field evaluation reveals huanglongbing resistance in citrus relatives. Plant Dis. 100, 1858–1869. doi: 10.1094/PDIS-03-16-0271-RE 30682983

[B59] Ramadugu C. Keremane M. McCollum T. G. Hall D. G. Roose M. L. (2016a). Developing resitance to HLB. Citrograph 7, 46–51. Available at: https://citrus-research-board-static.sfo2.digitaloceanspaces.com/citrograph/pdf/CRB-Citrograph-Mag-Q2-2016-web.pdf (Accessed July 25, 2024).

[B60] Ramadugu C. Roose M. L. (2024). Breeding HLB-resistant citrus and field evaluation of novel hybrids. Citrograph 15, 52–56. Available at: https://citrus-research-board-static.sfo2.digitaloceanspaces.com/citrograph/pdf/CRB-Citrograph-Mag-Q3-Summer-2024-Web.pdf (Accessed July 25, 2024).

[B61] Ratnaparkhe M. B. Wang X. Li J. Compton R. O. Rainville L. K. Lemke C.. (2011). Comparative analysis of peanut NBS-LRR gene clusters suggests evolutionary innovation among duplicated domains and erosion of gene microsynteny. New Phytol. 192, 164–178. doi: 10.1111/j.1469-8137.2011.03800.x 21707619

[B62] Rozas J. Ferrer-Mata A. Sánchez-DelBarrio J. C. Guirao-Rico S. Librado P. Ramos-Onsins S. E.. (2017). DnaSP 6: DNA sequence polymorphism analysis of large data sets. Mol. Biol. Evol. 34, 3299–3302. doi: 10.1093/molbev/msx248 29029172

[B63] Schwartz T. Nylinder S. Ramadugu C. Antonelli A. Pfeil B. E. (2015). The origin of oranges: A multi-locus phylogeny of rutaceae subfamily aurantioideae. Systematic Bot. 40, 1053–1062. doi: 10.1600/036364415X690067

[B64] Sharma R. Rawat V. Suresh C. G. (2017). Genome-wide identification and tissue-specific expression analysis of nucleotide binding site-leucine rich repeat gene family in Cicer arietinum (kabuli chickpea). Genom Data 14, 24–31. doi: 10.1016/j.gdata.2017.08.004 28840100 PMC5558467

[B65] Sievers F. Wilm A. Dineen D. Gibson T. J. Karplus K. Li W.. (2011). Fast, scalable generation of high-quality protein multiple sequence alignments using Clustal Omega. Mol. Syst. Biol. 7, 539. doi: 10.1038/msb.2011.75 21988835 PMC3261699

[B66] Singh K. Huff M. Liu J. Park J. W. Rickman T. Keremane M.. (2024). Chromosome-Scale, *De Novo*, Phased Genome Assemblies of Three Australian Limes: Citrus australasica, C. inodora and C. glauca. Plants (Basel) 13 (11), 1460. doi: 10.3390/plants13111460 PMC11174732 38891269

[B67] Steuernagel B. Witek K. Krattinger S. G. Ramirez-Gonzalez R. H. Schoonbeek H. J. Yu G.. (2020). The NLR-annotator tool enables annotation of the intracellular immune receptor repertoire. Plant Physiol. 183, 468–482. doi: 10.1104/pp.19.01273 32184345 PMC7271791

[B68] Stokes T. L. Kunkel B. N. Richards E. J. (2002). Epigenetic variation in Arabidopsis disease resistance. Genes Dev. 16, 171–182. doi: 10.1101/gad.952102 11799061 PMC155322

[B69] Sun J. Lu F. Luo Y. Bie L. Xu L. Wang Y. (2023). OrthoVenn3: an integrated platform for exploring and visualizing orthologous data across genomes. Nucleic Acids Res. 51, W397–W403. doi: 10.1093/nar/gkad313 37114999 PMC10320085

[B70] Takkouche A. Qiu X. Sedova M. Jaroszewski L. Godzik A. (2023). Unusual structural and functional features of TpLRR/BspA-like LRR proteins. J. Struct. Biol. 215, 108011. doi: 10.1016/j.jsb.2023.108011 37562586 PMC12244429

[B71] Tamura K. Stecher G. Kumar S. (2021). MEGA11: molecular evolutionary genetics analysis version 11. Mol. Biol. Evol. 38, 3022–3027. doi: 10.1093/molbev/msab120 33892491 PMC8233496

[B72] Tørresen O. K. Star B. Mier P. Andrade-Navarro M. A. Bateman A. Jarnot P.. (2019). Tandem repeats lead to sequence assembly errors and impose multi-level challenges for genome and protein databases. Nucleic Acids Res. 47, 10994–11006. doi: 10.1093/nar/gkz841 31584084 PMC6868369

[B73] van Wersch S. Li X. (2019). Stronger when together: clustering of plant NLR disease resistance genes. Trends Plant Sci. 24, 688–699. doi: 10.1016/j.tplants.2019.05.005 31266697

[B74] von Dahlen J. K. Schulz K. Nicolai J. Rose L. E. (2023). Global expression patterns of R-genes in tomato and potato. Front. Plant Sci. 14. doi: 10.3389/fpls.2023.1216795 PMC10641715 37965025

[B75] Wang L. Huang Y. Liu Z. He J. Jiang X. He F.. (2021). Somatic variations led to the selection of acidic and acidless orange cultivars. Nat. Plants 7 (7), 954–965. doi: 10.1038/s41477-021-00941-x 34140668

[B76] Wang Y. Zhou L. Li D. Dai L. Lawton-Rauh A. Srimani P. K.. (2015). Genome-wide comparative analysis reveals similar types of NBS genes in hybrid Citrus sinensis genome and original Citrus clementine genome and provides new insights into non-TIR NBS genes. PloS One 10, e0121893. doi: 10.1371/journal.pone.0121893 25811466 PMC4374887

[B77] Wang Y. Zhou L. Yu X. Stover E. Luo F. Duan Y. (2016). Transcriptome profiling of huanglongbing (HLB) tolerant and susceptible citrus plants reveals the role of basal resistance in HLB tolerance. Front. Plant Sci. 7. doi: 10.3389/fpls.2016.00933 PMC4923198 27446161

[B78] Weber K. C. Mahmoud L. M. Stanton D. Welker S. Qiu W. Grosser J. W.. (2022). Insights into the mechanism of Huanglongbing tolerance in the Australian finger lime (Citrus australasica). Front. Plant Sci. 13. doi: 10.3389/fpls.2022.1019295 PMC9634478 36340410

[B79] Wheeler T. J. Eddy S. R. (2013). nhmmer: DNA homology search with profile HMMs. Bioinformatics 29, 2487–2489. doi: 10.1093/bioinformatics/btt403 23842809 PMC3777106

[B80] Wu G. A. Prochnik S. Jenkins J. Salse J. Hellsten U. Murat F.. (2014). Sequencing of diverse mandarin, pummelo and orange genomes reveals complex history of admixture during citrus domestication. Nat. Biotechnol 32 (7), 656–662. doi: 10.1038/nbt.2906 24908277 PMC4113729

[B81] Wu G. A. Terol J. Ibanez V. López-García A. Pérez-Román E. Borredá C.. (2018). Genomics of the origin and evolution of Citrus. Nature 554, 311–316. doi: 10.1038/nature25447 29414943

[B82] Xie J. Chen Y. Cai G. Cai R. Hu Z. Wang H. (2023). Tree Visualization By One Table (tvBOT): a web application for visualizing, modifying and annotating phylogenetic trees. Nucleic Acids Res. 51, W587–w592. doi: 10.1093/nar/gkad359 37144476 PMC10320113

[B83] Yang S. Feng Z. Zhang X. Jiang K. Jin X. Hang Y.. (2006). Genome-wide investigation on the genetic variations of rice disease resistance genes. Plant Mol. Biol. 62, 181–193. doi: 10.1007/s11103-006-9012-3 16915523

[B84] Yang S. Zhang X. Yue J. X. Tian D. Chen J. Q. (2008). Recent duplications dominate NBS-encoding gene expansion in two woody species. Mol. Genet. Genomics 280, 187–198. doi: 10.1007/s00438-008-0355-0 18563445

[B85] Yin T. Han P. Xi D. Yu W. Zhu L. Du C.. (2023). Genome-wide identification, characterization, and expression profile ofNBS-LRRgene family in sweet orange (Citrussinensis). Gene 854, 147117. doi: 10.1016/j.gene.2022.147117 36526123

[B86] Zhai C. Zhang Y. Yao N. Lin F. Liu Z. Dong Z.. (2014). Function and interaction of the coupled genes responsible for Pik-h encoded rice blast resistance. PloS One 9, e98067. doi: 10.1371/journal.pone.0098067 24896089 PMC4045721

[B87] Zhang M. Coaker G. (2017). Harnessing effector-triggered immunity for durable disease resistance. Phytopathology 107, 912–919. doi: 10.1094/PHYTO-03-17-0086-RVW 28430023 PMC5810938

[B88] Zhang Y. Guo M. Shen J. Song X. Dong S. Wen Y.. (2019). Comparative genomics analysis in grass species reveals two distinct evolutionary strategies adopted by R genes. Sci. Rep. 9, 10735. doi: 10.1038/s41598-019-47121-8 31341223 PMC6656885

[B89] Zhu S. Li Y. Vossen J. H. Visser R. G. Jacobsen E. (2012). Functional stacking of three resistance genes against Phytophthora infestans in potato. Transgenic Res. 21, 89–99. doi: 10.1007/s11248-011-9510-1 21479829 PMC3264857

