# Supplementary material for: Deep R-gene discovery in HLB resistant wild Australian limes uncovers evolutionary features and potentially important loci for hybrid breeding
Source: Front Plant Sci. 2025 Jan 29;15:1503030. doi: 10.3389/fpls.2024.1503030 (PMC11831368; doi:10.3389/fpls.2024.1503030)

**Fig. S1. A** Bar graph of similarity summary between genomes of *C. sinensis* and *C. clementina.* Percentages in the second column represent sums of the projections of the matches of *C. sinensis* on *C. clementina* per similarity percentile divided by the total length of the latter. **B** Dot plot showing synthetic similarity between genomes of *C. clementina* (Horizontal) and *C. sinensis* (Vertical). **C** Rearrangement of the chromosome numbers in *C. sinensis* after contigs were sorted, leading to chromosome renaming as: Chr1→Chr7, Chr3→Chr5, Chr4→Chr1, Chr5→Chr3, Chr7→Chr4.


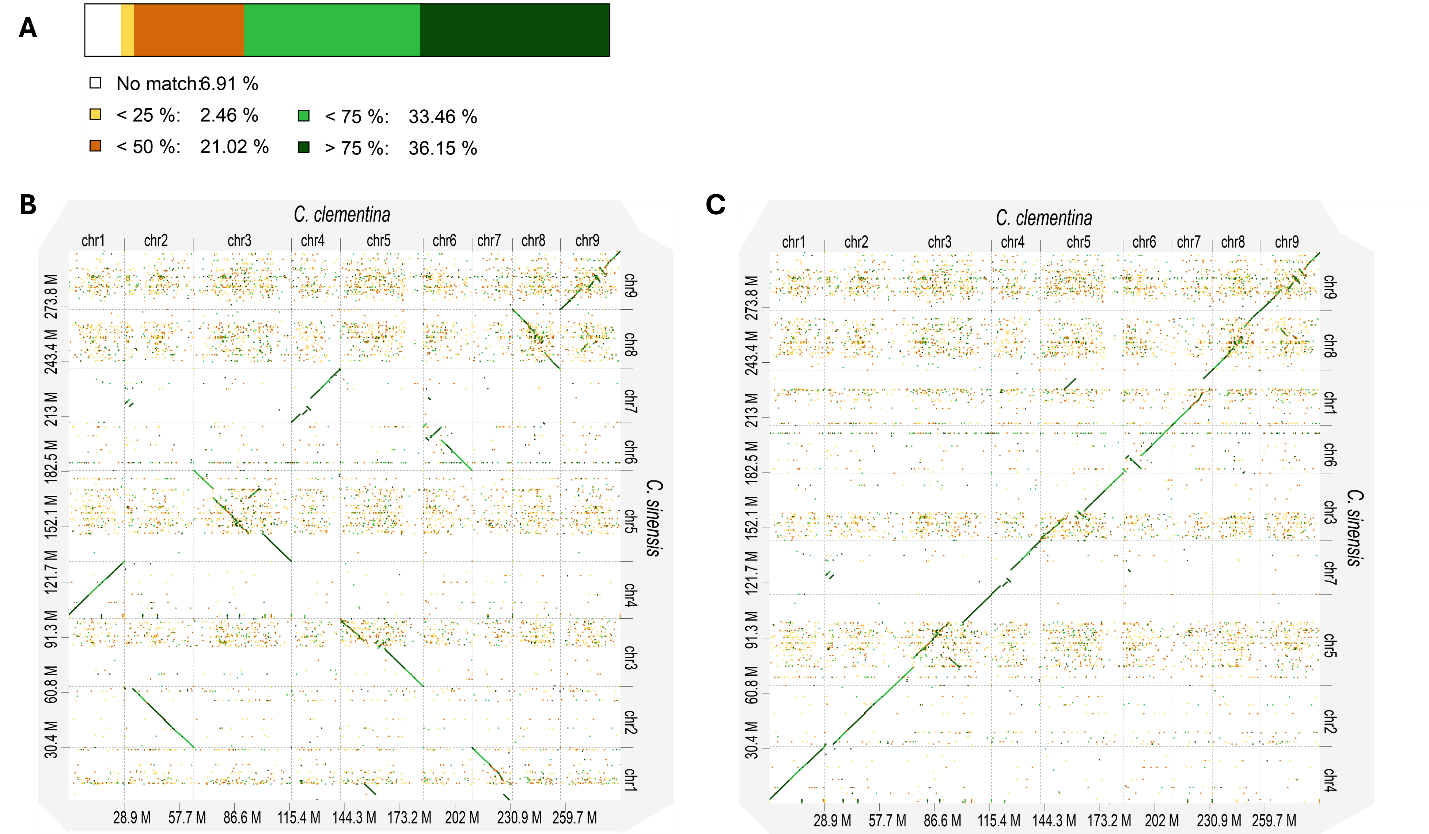


**Table S1.** List of Pfam accessions used in R-gene identification and classification by FindPlantNLR.


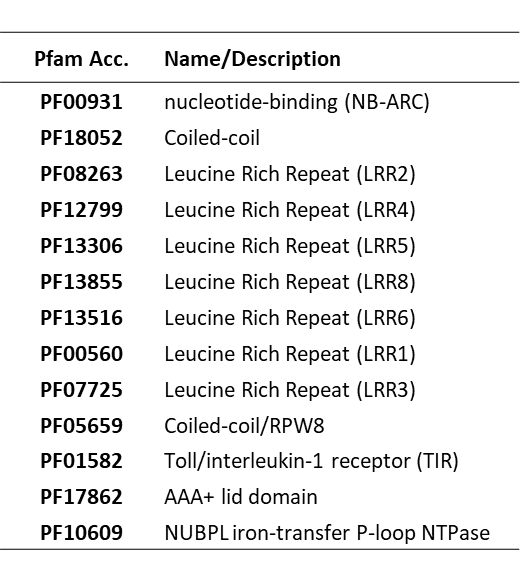


**Table S2.** Number of NBARC-genes from the five *Citrus* species that were identified to match the genome annotation transcripts using program GffCompare (Pertea and Pertea, 2020). The specific matching relationship of NBARC-genes to the genome transcripts are classified by single letter codes as follows: **c**, contained in reference (intron compatible); **e**, single exon that partially covers reference intron; **i**, fully contained within a reference intron; **j**, multi-exon with one or more junction match; **k**, reverse containment; **m**, full retainment of intron(s); **n**, partial retainment of intron(s); **o**, other same strand overlap with reference exons; **q**, complete and exact match to the reference; **x**, exonic overlap on opposite strand; **y**, contains a reference within its intron(s). Refer to Pertea and Pertea (2020) for a visual description of the different transcript classification codes.

**
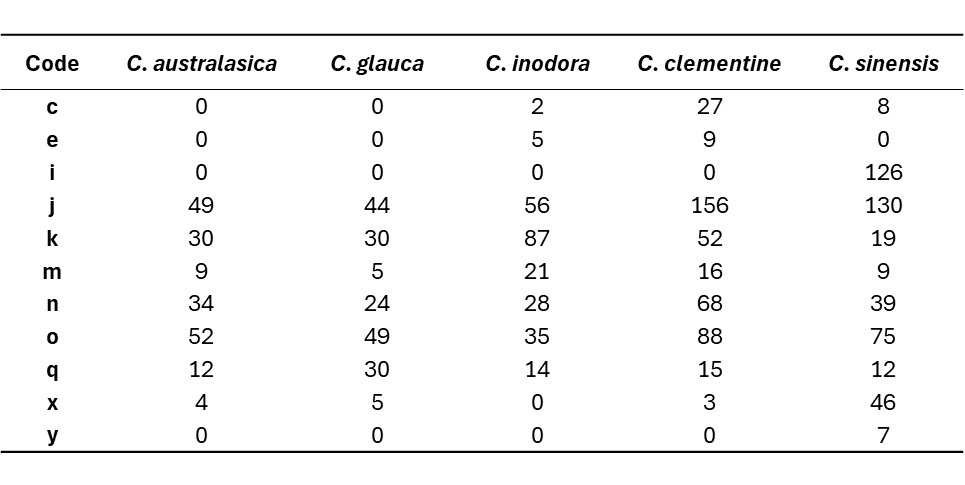
**

**Figure. S2.** Pairwise chromosomal synteny between citrus genomes on chromosomes (chromosome sizes not depicted to proportion. **(A)** Number of pairwise ortholog genes (row/column, **(B)** *C. clementina* vs. *C. sinensis* , **(C)** Within Australian limes, **(D)** *C. clementina* vs. Australian limes, and **E** *C. sinensis* vs*.* Australian limes


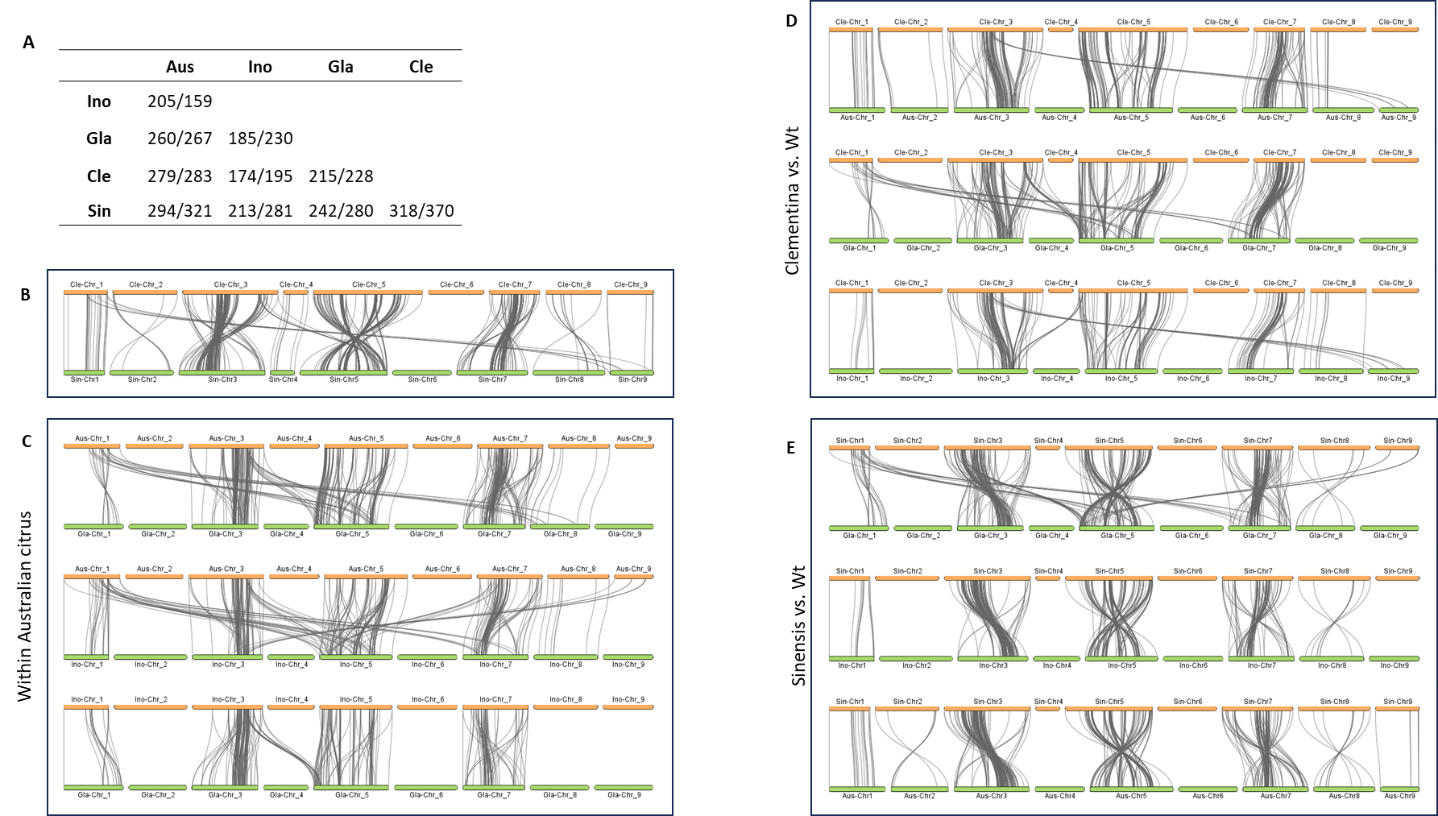


**Table S6.** Genes from *C. clementina* and *S. sinensis* that have Ka/Ks values > 1 in comparison to Australian limes. Sequence blast and GO annotation were conducted at https://www.citrusgenomedb.org/


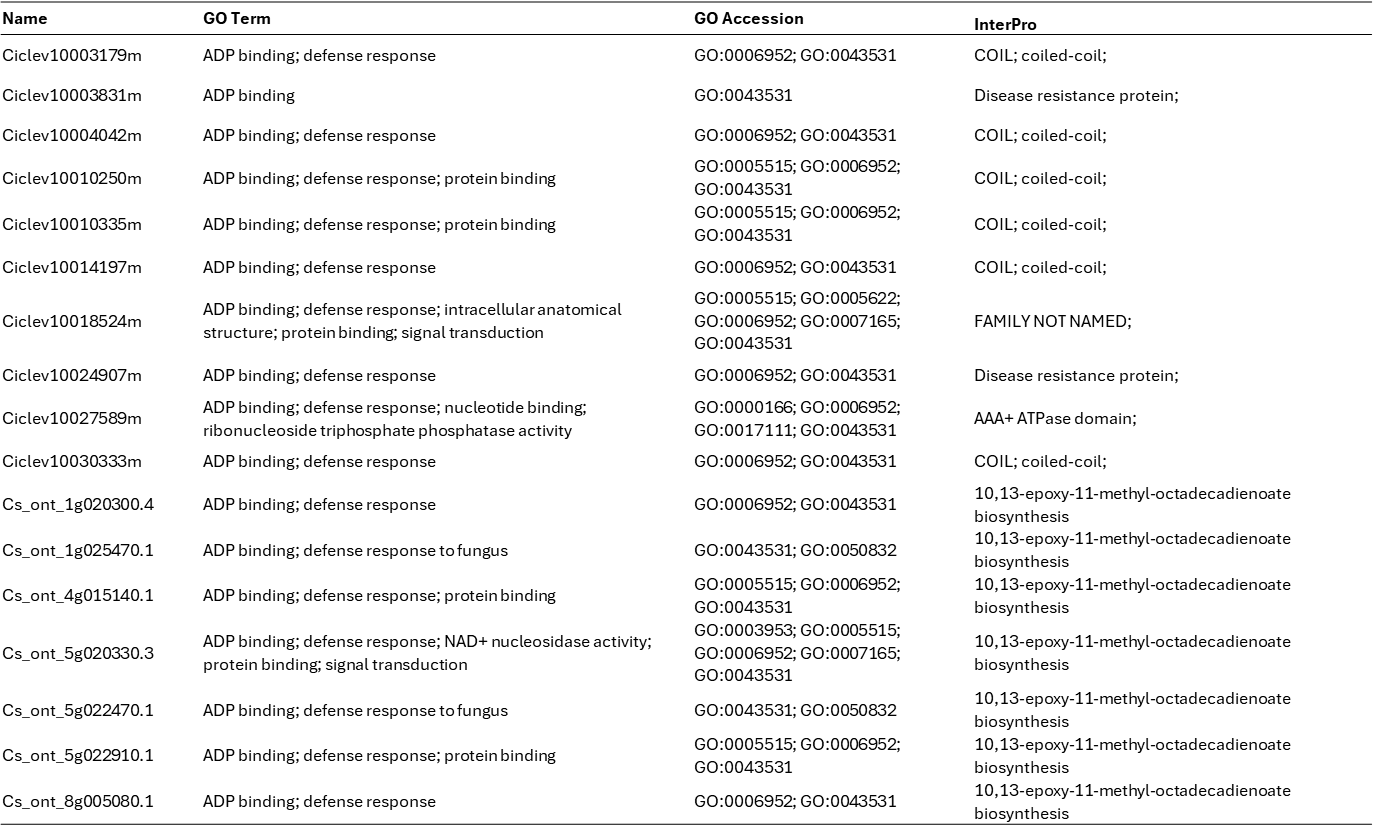

Supplement: Supplementary file 1 [file SupplementaryFile1.docx]
